# Impaired Secondary Platelet Response in Chronic Kidney Disease as a Consequence of Prior Platelet Activation

**DOI:** 10.1016/j.jacbts.2025.101355

**Published:** 2025-08-05

**Authors:** Constance C.F.M.J. Baaten, Julia Wollenhaupt, Tobias M. Henning, Sonja Vondenhoff, Jonas R. Schröer, Eleni Stamellou, Turgay Saritas, Berkan Kurt, Leonard Boger, Alessandra Antwerpen, Juliane Hermann, Magdolna Nagy, Marieke Sternkopf, Eva Miriam Buhl, Ute Raffetseder, Paola E.J. van der Meijden, Marijke J.E. Kuijpers, Henri M.H. Spronk, Stefan J. Schunk, Joachim Jankowski, Danilo Fliser, Thimoteus Speer, Peter Boor, Rafael Kramann, Florian Kahles, Jürgen Floege, Nikolaus Marx, Heidi Noels

**Affiliations:** a Institute for Molecular Cardiovascular Research (IMCAR), University Hospital RWTH Aachen, Aachen, Germany; b Department of Biochemistry, Cardiovascular Research Institute Maastricht, Maastricht University, Maastricht, the Netherlands; c Aachen-Maastricht Institute for Cardiorenal disease (AMICARE), Aachen, Germany; d Department of Nephrology and Clinical Immunology, University Hospital RWTH Aachen, Aachen, Germany; e Department of Nephrology, University Hospital of Ioannina, Ioannina, Greece; f Department of Internal Medicine, Nephrology and Transplantation, Erasmus Medical Center, Rotterdam, the Netherlands; g Department of Internal Medicine I, University Hospital RWTH Aachen, Aachen, Germany; h Institute of Pathology, University Hospital RWTH Aachen, Aachen, Germany; i Electron Microscopy Facility, University Hospital RWTH Aachen, Aachen, Germany; j Thrombosis Expertise Center, Heart and Vascular Center, Maastricht University Medical Center+, Maastricht, the Netherlands; k Department of Internal Medicine, Maastricht University Medical Center+, Maastricht, the Netherlands; l Department of Internal Medicine IV, Nephrology and Hypertension, Saarland University, Homburg/Saar, Germany; m Department of Pathology, Cardiovascular Research Institute Maastricht, Maastricht University Medical Centre, Maastricht, the Netherlands; n Department of Internal Medicine IV, Goethe University Frankfurt, Frankfurt am Main, Germany

**Keywords:** acetylsalicylic acid, bleeding, chronic kidney disease, platelets, thrombosis

## Abstract

Patients with chronic kidney disease (CKD) are at increased risk of thrombotic and hemorrhagic complications. Findings on platelet defects in CKD are conflicting. Therefore, we examined platelet function in CKD stage 3 to 5 dialysis patients without antithrombotic therapy, in CKD5D/hemodialysis patients on acetylsalicylic acid (ASA) as well as in a CKD mouse model. Patients with advanced CKD without antithrombotic therapy show platelet preactivation with a partial secondary platelet dysfunction mainly upon collagen/GPVI stimulation. Platelets from hemodialysis patients on ASA showed a less severe CKD-associated secondary platelet dysfunction compared with those not taking ASA, with comparable observations in CKD mice on ASA vs vehicle.

## Highlights

• Platelet dysfunction has been reported in CKD, although without a focused analysis of the inherent impact of CKD on platelet function in the absence of antithrombotic therapy.
• Platelets of patients with advanced CKD who are without antithrombotic therapy show signs of prior platelet activation that led to a secondary reduced platelet function.
• Secondary reductions in platelet function increase with CKD severity but appear to be less pronounced in patients who are on acetylsalicylic acid.

## Visual Abstract

**Figure:**
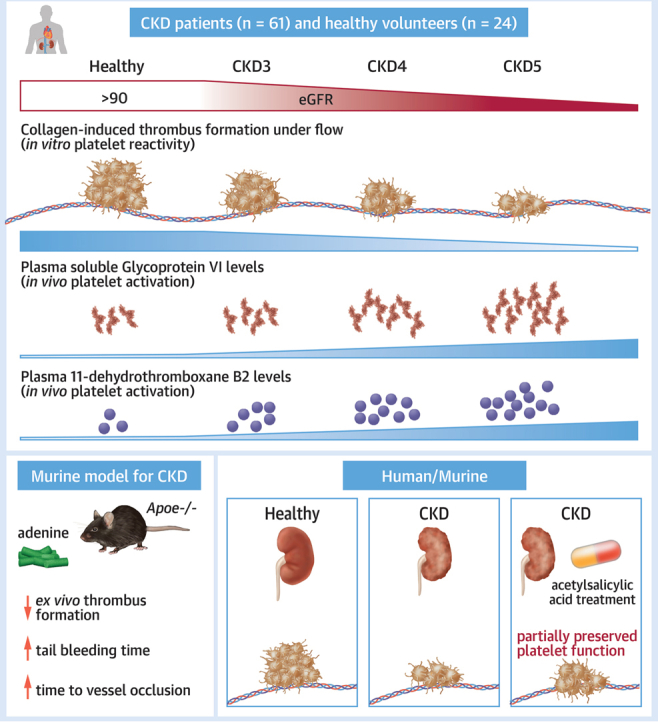

Platelets are central to hemostasis and a balanced platelet response to vascular injury is key in the cessation of bleeding, while preventing a thrombotic response.^1^ In chronic kidney disease (CKD), this balance is lost, and both thrombotic and hemorrhagic complications increase.^2^, ^3^, ^4^ Platelet function in CKD has been the topic of previous investigations; however, conflicting results are reported ranging from a decreased platelet response to a normal or even enhanced platelet reactivity.^5^, ^6^, ^7^, ^8^, ^9^ CKD is a chronic, progressive disease that affects 13.4% of the global population. It is classified in stages based on the reduction in kidney function, where CKD stages 1 to 2 represent a normal-to-mild reduction in function, CKD3 a mild-to-moderate reduction, and CKD4 (advanced CKD) a severe reduction. CKD5D indicates stage 5 kidney failure requiring dialysis.^10^ The variety in the degree of CKD kidney dysfunction, but also in CKD etiology, coexisting comorbidities, and treatments (which may include antiplatelet and anticoagulation therapy) add to the complexity of potential pathophysiological effects on platelets and may thus contribute to the variability in reported platelet function in CKD.

To provide further insights into platelet impairment in CKD, we examined the platelet response of patients with CKD of varying severity using an ex vivo whole-blood thrombus formation assay under arterial flow conditions. We specifically aimed to focus on the inherent impact of CKD on platelet responses without interference of anticoagulant or antiplatelet therapy and therefore applied a strict selection of patients who were not on any anticoagulant or antiplatelet therapy. In addition, since CKD patients are often treated with acetylsalicylic acid (ASA) (better known as aspirin), an antiplatelet drug to prevent secondary atherothrombotic complications we also included 1 CKD patient group on ASA. Findings were compared with ex vivo and in vivo measurements of thrombosis and bleeding in a murine CKD model with vs without ASA treatment.

## Methods

A detailed Materials and Methods section is available in the Supplemental Appendix.

### Patient study

Experiments were approved by the local Medical Ethics Committee (EK040/19 and EK377/19, University Hospital Aachen). Written informed consent was obtained conform the Declaration of Helsinki.

#### CKD patients and healthy control subjects

The main platelet phenotyping was performed in blood samples from CKD patients reporting at the nephrology department of the University Hospital RWTH Aachen from June 2019 until May 2024 and fulfilling the inclusion criteria. Patients were eligible for participation when they were ≥18 years of age and diagnosed with CKD stage 3 or higher, where indicated being on hemodialysis (CKD5D). With our focus on the inherent impact of CKD on platelets without the interference of antiplatelet therapy, patients were not eligible for study participation when treated with antiplatelet or anticoagulant medication up to 14 days before blood collection, apart from 1 study group including specifically CKD5D patients on ASA treatment, where indicated. Furthermore, patients were not eligible for study participation in case of a hemoglobin value <10 g/dL, an active infection, malignancy, or prior hematological malignancy. CKD5D patients donated blood at the end of the longest interdialytic interval, just before the start of the hemodialysis session and before the administration of heparin. CKD5D patients were only eligible for study participation when they had an arteriovenous fistula instead of a venous catheter for vascular access and blood sampling (to prevent interference of the anticoagulant lock associated with the latter). All patients were hemodynamically stable. Healthy volunteers reported not taking any antithrombotic medication in the past 14 days before blood withdrawal.

#### Cardiovascular disease patients with healthy kidney function

For selected readouts, a group of patients with cardiovascular disease (CVD) but with healthy kidney function was recruited by the cardiology department of the hospital (from August 2023 until May 2024). The included patients were hospitalized and ambulatory cardiological patients, including patients with atherosclerotic CVD, heart failure, and valvular heart disease with cardiovascular risk factors and cardiometabolic comorbidities present, with additional inclusion criteria being hemodynamically stable and ≥18 years of age. The same exclusion criteria were applied as for the CKD patients, being a hemoglobin value <10 g/dL, an active infection, malignancy, or prior hematological malignancy. Also, patients on antiplatelet or anticoagulant medication up to 14 days before blood collection were not eligible for study participation, apart from 1 study group specifically on ASA treatment.

#### CARE FOR HOMe cohort

The CARE FOR HOMe (Cardiovascular And REnal outcome in CKD stage 2–4 patients—The FOuRth HOMburg evaluation) cohort was used as a validation cohort to analyze plasma levels of soluble glycoprotein VI (GPVI). This cohort contains clinically stable patients with confirmed CKD (analyzed biosamples and associated glomerular filtration rate/CKD stage from 1 year of follow-up).^11^ In short, inclusion criteria include CKD stages 2 to 4 and ≥18 years of age, whereas main exclusion criteria included active malignancy, clinically apparent infections, active immunosuppressive therapy, kidney transplant, and acute kidney injury.^11^^,^^12^ The collection and preparation of plasma samples for the CARE FOR HOMe study has been published previously.^11^

### Mice

All animal experiments were approved by the local authorities (LANUV, Nr. 81-02.04.2017.A504) and complied with the local, national, and European Union ethical guidelines. Adenine nephropathy was induced by feeding male C57BL/6J apolipoprotein E–deficient (*Apoe*^−/−^) mice a high-fat diet for 4 weeks followed by a high-fat, adenine-rich diet for 14 days.^13^ In parallel, control mice were fed the high-fat diet without adenine. Where indicated, control and adenine-fed mice were given ASA via the drinking water (75 mg/L ASA) during the last 7 days. At the end of the experiment, in vivo thrombosis, tail bleeding, or thrombus formation under flow was studied.

### Statistics

Continuous data are presented as the median with 25th to 75th percentiles (Q1-Q3) or mean ± SD. Normality of the data was assessed using the Shapiro-Wilk test. Two groups were compared using Student’s *t*-test or Mann-Whitney *U* test, according to distribution. More than 2 groups were compared using analysis of variance with Sídák’s or Dunnett’s post hoc test or Kruskal-Wallis test with Dunn’s post hoc test. Paired data were analyzed using repeated measures analysis of variance with Sídák’s post hoc test. Association of 2 continuous variables was assessed using linear regression and either Pearson’s or Spearman’s correlation coefficient (*r*). Statistical analyses were performed using GraphPad Prism version 10 (GraphPad Software), and a *P* value <0.05 was considered statistically significant.

## Results

### Impairment of collagen (GPVI)-mediated platelet activation in patients with advanced CKD

Supplemental Tables S1 to S3 display the patient characteristics and hematological parameters. CKD3 patients showed an increased number of leukocytes compared with the healthy control group. The erythrocyte number and hemoglobin level were significantly decreased in patients with CKD4, CKD5D, and CKD5D ASA (Supplemental Table S1). A significant decrease in platelet number was observed only in patients with CKD5D. Patients in different CKD stages did not display considerable differences in terms of medication (antihypertensive agents, glucose lowering, immune suppressants), except for increased use of erythropoietin in CKD5D and CKD5D ASA, and lipid-lowering medication in CKD5D ASA.

**Figure 1:**
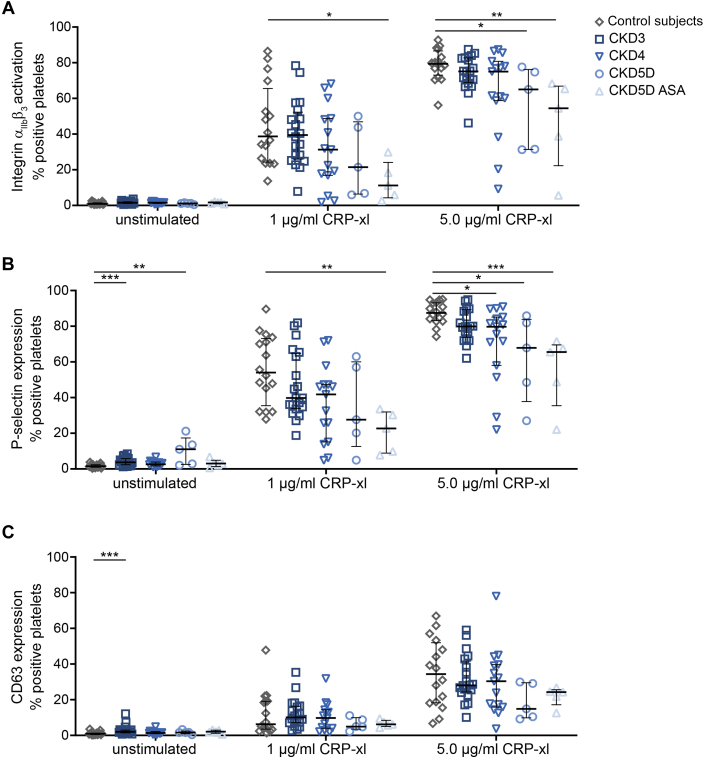
Reduced CRP-XL–Induced Integrin α_IIb_β_3_ Activation and P-Selectin Expression in Platelets of CKD5D Patients Isolated platelets (50 × 10^6^/mL) were left unstimulated or activated with collagen-related peptide, crosslinked (CRP-XL) for 15 minutes. Integrin α_IIb_β_3_ activation (A), P-selectin expression (B), and CD63 expression (C) were assessed using the FITC-conjugated PAC-1 antibody, a PE (phycoerythrin)-conjugated anti–P-selectin antibody or an APC (allophycocyanin)-conjugated anti-CD63 antibody, respectively. Values are median with 25th-75th percentiles. ∗*P* < 0.05; ∗∗*P* < 0.01; ∗∗∗*P* < 0.001 (Kruskal Wallis test with Dunn’s post hoc test). Healthy control subjects n = 16-18, chronic kidney disease stage 3 (CKD3) n = 20, CKD4 n = 15, chronic kidney disease stage 5 dialysis (CKD5D) n = 5, and CKD5D acetylsalicylic acid (ASA) n = 5.

An analysis of isolated platelets by flow cytometry revealed signs of weak granule secretion (P-selectin and/or CD63 expression) in unstimulated platelets from CKD3 and CKD5D patients (Figure 1). Upon stimulation with collagen-related peptide, crosslinked (CRP-XL), triggering the GPVI signaling pathway, impairments could be detected in terms of reduced integrin activation (CKD5D and CKD5D ASA) and P-selectin expression (CKD4, CKD5D, and CKD5D ASA) in comparison with control subjects (Figure 1). Also, a significant reduction in thrombin receptor activating peptide 6 (TRAP-6)-induced P-selectin expression (signaling via PAR1 receptor) was observed in CKD5D and CKD5D ASA, whereas adenosine diphosphate (ADP)-induced platelet activation (P2Y_1_ and P2Y_12_ mediated) was not significantly altered (Supplemental Figure S1).

In diluted whole blood, resting platelets from CKD patients showed no signs of integrin α_IIb_β_3_ activation or P-selectin expression (Supplemental Figure S2). Again, platelets from CKD4 and CKD5D ASA patients showed a significantly decreased integrin response upon GPVI stimulation with CRP-XL in comparison with healthy platelets (Supplemental Figure S2), whereas no differences in platelet reactivity between CKD patients and healthy control subjects were found when platelet activation was induced by ADP or TRAP-6 (Supplemental Figure S2).

In summary, patients with advanced CKD display an impairment in platelet activation upon stimulation of the collagen receptor GPVI.

### Decline in ex vivo thrombus formation and fibrin formation with progression of CKD

**Figure 2:**
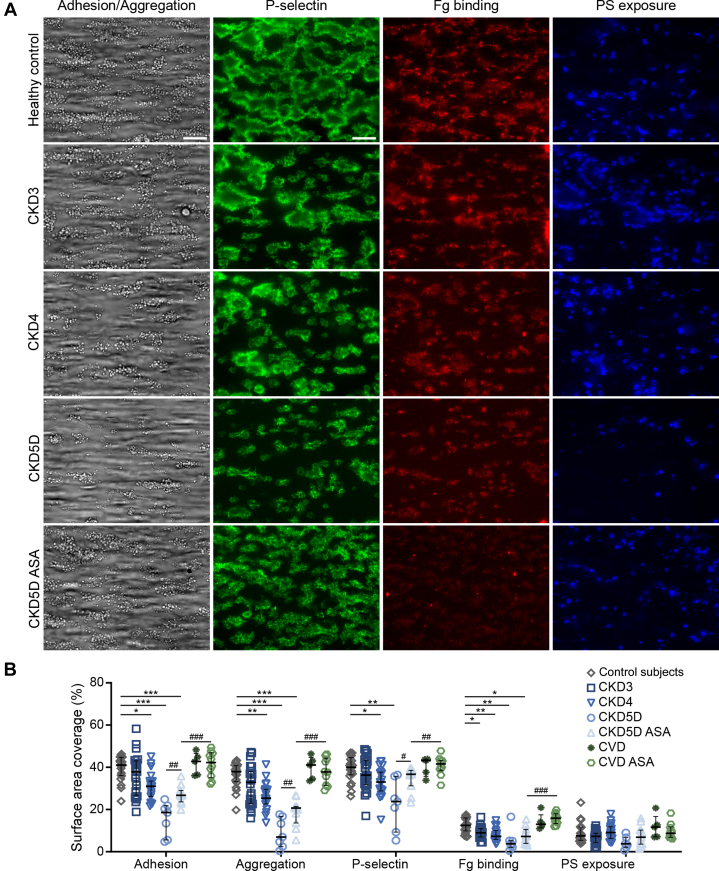
Reduced Ex Vivo Platelet Adhesion and Aggregation Under Flow With Increasing CKD Progression Citrate anticoagulated whole blood of healthy control subjects or CKD patients was recalcified in the presence of PPACK and perfused over a collagen type I surface at 1,000 s^−1^ for 4 minutes. Integrin activation was monitored by determining the amount of bound fluorescent fibrinogen (Fg) to the thrombi. P-selectin expression and phosphatidylserine (PS) exposure were measured by labeling the formed thrombi with an antibody directed against P-selectin and annexin A5, respectively. (A) Representative images of adhesion and aggregation, P-selectin expression, fibrinogen binding and PS exposure. Scale bar = 20 μm. (B) Quantification of the surface area covered by adhered platelets, aggregated platelets, P-selectin–positive platelets, bound fibrinogen, and PS-positive platelets. Values are median with 25th-75th percentiles. ∗*P* < 0.05; ∗∗*P* < 0.01; ∗∗∗*P* < 0.001 (PS exposure, CKD5D vs healthy control subjects *P* = 0.056): Kruskal Wallis test with Dunn’s post hoc test for comparison vs healthy control group. ^#^*P* < 0.05; ^##^*P* < 0.01; ^###^*P* < 0.001 Mann-Whitney *U* test for comparison of CKD5D vs CKD5D ASA. Healthy control subjects n = 21, CKD3 n = 24, CKD4 n = 17, CKD5D n = 7, CKD5D ASA n = 11, cardiovascular disease (CVD) n = 5 and CVD ASA n = 10. Abbreviations as in Figure 1.

To assess the hemostatic consequences of reduced integrin activation and P-selectin expression of CKD platelets, thrombus formation was studied ex vivo under arterial flow conditions. Platelet adhesion and aggregation were significantly reduced in CKD4/CKD5D, correlating moderately with eGFR (Figure 2, Supplemental Figure S3A). Further, platelets that were incorporated into platelet aggregates showed a significantly reduced P-selectin expression and fibrinogen binding, the latter indicative of decreased integrin activation. Also, a significant reduction in procoagulant activity (as measured by phosphatidyl serine [PS] exposure^14^) was observed in thrombus-incorporated platelets in CKD5D. Remarkably, compared with CKD5D, platelets from CKD5D ASA patients showed a less severe CKD5D-associated decline in secondary ex vivo thrombus formation relative to healthy control subjects (Figure 2). Instead, the addition of ASA to blood ex vivo did not affect whole-blood thrombus formation (Supplemental Figure S4), in line with previous studies analyzing the impact of ex vivo supplementation of ASA in these assays.^15^ This indicates that the observed difference in thrombus formation between CKD5D ASA and CKD5D patients in our ex vivo flow assay is not caused by a direct effect of ASA, but instead is due to a prior in vivo impact of ASA. Thus, this suggests that in CKD5D ASA patients, ASA-mediated protection from prior platelet activation induced by CKD5D conditions contributes to their partially reduced platelet dysfunction in ex vivo thrombus formation compared with CKD5D patients. However, despite this partial preservation of platelet responses in CKD5D ASA patients, platelet aggregation and fibrinogen binding responses in CKD5D ASA patients remained significantly lower than those in healthy control subjects (Figure 2). By comparison, patients with CVD, but with healthy kidneys, did not display differences in ex vivo thrombus formation capacity relative to healthy control subjects, and neither were platelet responses different between patients without vs with ASA treatment (Figure 2, Supplemental Tables S1 and S3).

**Figure 3:**
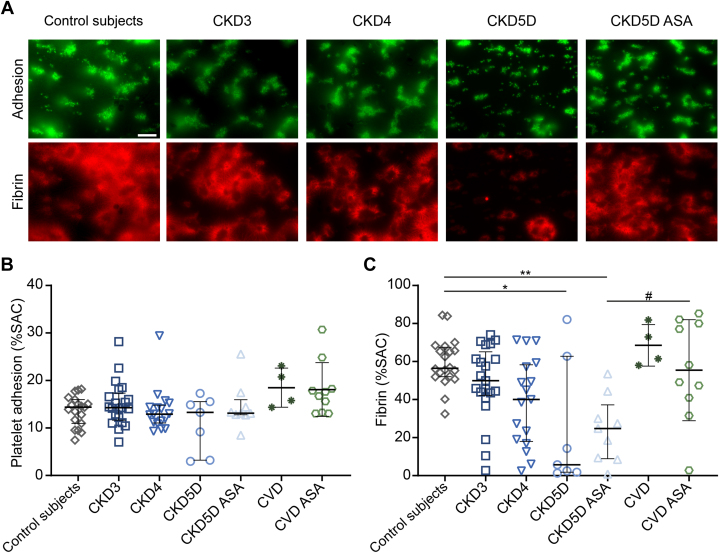
Impaired Platelet-Mediated Fibrin Formation Under Flow in CKD5 Patients on Hemodialysis Using a co-perfusion system, citrate anticoagulated blood of healthy control subjects or CKD patients was recalcified and subsequently perfused over a collagen/tissue factor (TF) surface for 10 minutes at 1,000 s^−1^ allowing the formation of thrombi and fibrin. (A) Representative fluorescence images of thrombus (DiOC6-labeled platelets) and fibrin formation after 10 minutes of blood perfusion. Scale bar = 20 μm. (B and C) Quantification of the platelet (B) and fibrin (C) surface area coverage after 10 minutes of perfusion. Values are median with 25th-75th percentiles. ∗*P* < 0.05; ∗∗*P* < 0.01 (fibrin, CKD4 vs healthy control subjects *P* = 0.074) Kruskal Wallis test with Dunn’s post hoc test for comparison vs healthy control group. ^#^*P* < 0.05 (Mann-Whitney *U* test for comparison of CKD5D vs CKD5D ASA). Healthy control subjects n = 20, CKD3 n = 21, CKD4 n = 17, CKD5D n = 7, CKD5D ASA n = 9, CVD n = 4, and CVD ASA n = 10. HD = hemodialysis; SAC = surface area coverage; other abbreviations as in Figures 1 and 2.

To study the effect of CKD on the ability of platelets to support fibrin formation under flow, whole blood perfusion experiments were repeated using a collagen/tissue factor (TF) surface, allowing thrombin generation. Although no differences in platelet surface area coverage were observed under these coagulating conditions (Figures 3A and 3B), platelet-dependent fibrin formation was significantly impaired in CKD4 and in both CKD5D and CKD5D ASA (Figures 3A and 3C). Again, CVD patients with healthy kidney function did not show differences compared to healthy control subjects (Figures 3A and 3C).

In summary, CKD progression is associated with a decline in ex vivo platelet-dependent thrombus formation and fibrin formation capacity in whole blood under flow. Since this decline is at least in part less severe in CKD5D ASA patients compared with CKD5D without ASA treatment, our findings suggest a prior preactivation with partial secondary functional impairment of platelets in CKD5D patients, which is less severe in CKD5D ASA patients.

### CKD-induced defects in ex vivo thrombus formation cannot be reversed by restoration of the erythrocyte number but are partly caused by uremic toxin accumulation

Anemia is common in CKD and affects thrombus formation, the latter because erythrocytes support platelet margination toward the vessel wall and thereby platelet adhesion and aggregation.^4^^,^^16^ We found a significant correlation of hemoglobin levels with platelet adhesion and aggregation (Supplemental Figure S3B). However, supplementation of autologous or healthy erythrocytes (blood type O^−^) to blood from CKD4 patients only enhanced P-selectin expression but did not result in an overall increase in thrombus formation (Supplemental Figures S5A and S5B, Supplemental Table S4). Vice versa, whole-blood perfusion experiments in which healthy platelets were reconstituted with CKD4 plasma and erythrocytes could not mimic the impairment in thrombus formation seen in CKD4 (Supplemental Figure S5C). Furthermore, reconstitution of CKD4 platelets with healthy plasma and erythrocytes could not rescue the defect in thrombus formation (Supplemental Figure S5C). Overall, this suggests that the CKD-induced platelet defects accumulate over time, are irreversible, and cannot be reversed by a correction of anemia.

**Figure 4:**
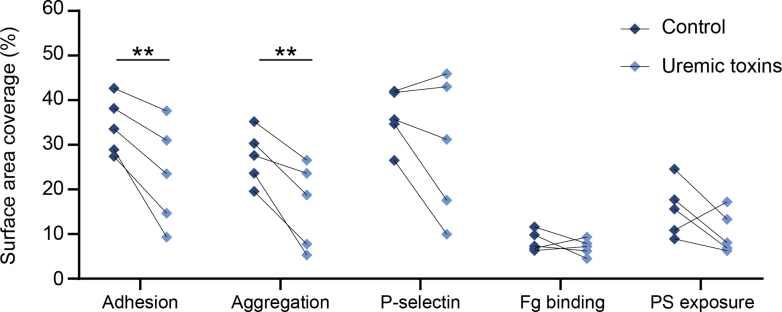
Short-Term Exposure to Uremic Toxins Causes an Impairment of Platelet Adhesion and Aggregation Under Flow Citrate anticoagulated whole blood of healthy control subjects was treated with a mixture of phenylacetic acid, indoxyl sulphate, hippuric acid, kynurenic acid, p-cresyl sulphate, methylguanidine, and guanidinosuccinic acid, at concentrations reflecting advanced CKD,^46^ or alternatively, with vehicle control. After a 5-minute incubation at room temperature, the blood was recalcified in the presence of PPACK and perfused over a collagen type I surface at 1,000 s^−1^ for 4 minutes. Integrin activation was assessed by the amount of bound fluorescent fibrinogen. After blood perfusion, P-selectin expression and PS exposure were detected by perfusing buffer with an anti–P-selectin antibody and annexin A5, respectively. Quantification of the surface area covered by adhered platelets, aggregated platelets, P-selectin–positive platelets, bound fibrinogen, and PS-positive platelets. ∗∗*P* < 0.01 (repeated measures 2-way analysis of variance with Šídák’s post hoc test), n = 5. Abbreviations as in Figures 1 and 2.

Instead, incubation of healthy blood with a mixture of 7 of the most up-regulated uremic toxins at concentrations reflecting advanced CKD conditions did result in a significant decrease in platelet adhesion and aggregation under flow (Figure 4). Although the uremic toxin mix did not directly affect platelet activation marker expression (Supplemental Figure S6A), a trend towards a reduced platelet count upon toxin mix addition (Supplemental Figure S6B) and the occurrence of platelet aggregates in the blood observed in 90% of the uremic toxin–treated samples analyzed during whole-blood flow assays before reaching the collagen surface rather hinted towards a preactivated state of the platelets upon uremic toxin addition with subsequent loss of collagen-induced thrombus formation on site. Levels of the uremic toxins homocysteine and indoxyl sulfate were confirmed to be up-regulated in our CKD cohort, with the highest plasma levels of homocysteine and indoxyl sulfate in CKD4. However, levels of indoxyl sulfate in patients’ plasma showed no direct correlation with homocysteine levels (Supplemental Figure S7, Supplemental Table S5).

In summary, CKD-induced defects in ex vivo thrombus formation cannot be reversed by correction of anemia but are at least partly caused by uremic toxins.

### Elevated plasma levels of soluble GPVI in patients with severe renal dysfunction suggest prior platelet activation

**Figure 5:**
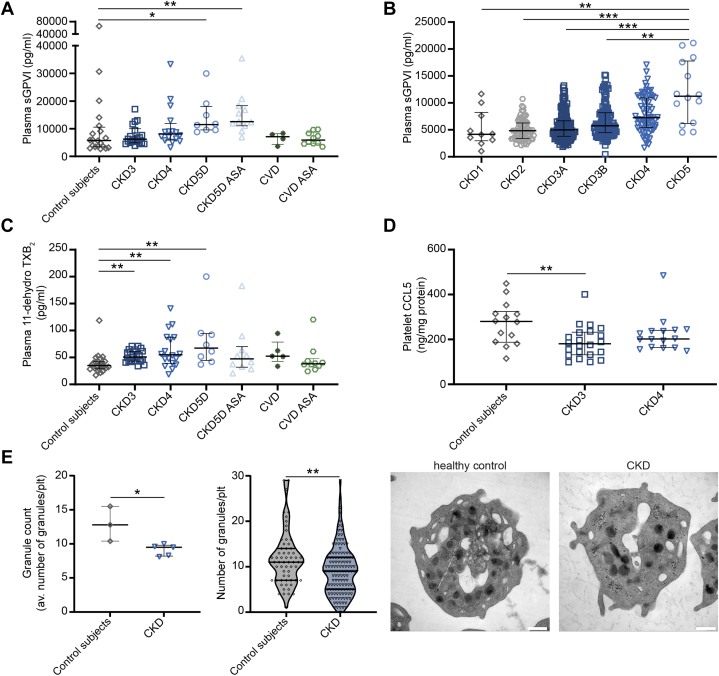
Plasma levels of soluble GPVI Increase as CKD Advances, Whereas the Number of Platelet Granules as Well as CCL5 Content Are Reduced in CKD (A and B) Plasma levels of soluble GPVI as measured by enzyme-linked immunosorbant assay (ELISA). (A) Healthy control subjects n = 20, CKD3 n = 22, CKD4 n = 16, CKD5D n = 8, CKD5D ASA n = 11, CVD n = 4, and CVD ASA n = 10; (B) independent CARE FOR HOMe cohort with a total of 420 patients. (C) Plasma levels of 11-dehydro thromboxane B_2_ as measured by ELISA, healthy control subjects n = 24, CKD3 n = 24, CKD4 n = 18, CKD5D n = 8, CKD5D ASA n = 10, CVD n = 5, and CVD ASA n = 10. (D) CCL5 levels in lysates of washed platelets of healthy control subjects (n = 14), CKD3 (n = 22), and CKD4 patients (n = 16) as determined by ELISA. (E) Average platelet granule count, number of granules per platelet, and representative images as analyzed by electron microscopy in platelets from 3 healthy control subjects and 5 CKD patients (CKD 4 n = 3, CKD5D ASA n = 2). Values are median with 25th-75th percentiles. ∗*P* < 0.05; ∗∗*P* < 0.01; ∗∗∗*P* < 0.001 (Kruskal Wallis test with Dunn’s post hoc test). Abbreviations as in Figures 1 and 2.

Platelet factor 4 (PF4) was significantly reduced in plasma of CKD5D patients, whereas glycocalicin—the soluble form of the GPIbα receptor (binding to von Willebrand factor)—was unaltered in CKD (Supplemental Figures S8A and S8B). However, levels of soluble GPVI (platelet collagen receptor, shed upon platelet activation) were elevated in CKD (but not in CVD patients with healthy kidney function) (Figure 5A). This could be confirmed in a large, independent cohort of CKD patients, displaying a gradual increase in soluble GPVI with CKD stage progression and significantly increased plasma levels of soluble GPVI in patients with CKD5 (Figure 5B). Moreover, we observed an increase in plasma levels of 11-dehydro thromboxane B2 (11-dehydro TXB_2_), a metabolite of thromboxane A2 (TXA_2_), in CKD3-5D patients compared with healthy control subjects, indicating increased TXA_2_ formation in CKD. No significant up-regulation of plasma 11-dehydro TXB_2_ was detected in CKD stage 3 to 5 dialysis patients on ASA (Figure 5C). Levels of sGPVI and 11-dehydro TXB2 did not correlate with plasma levels of neither homocysteine nor indoxyl sulfate (Supplemental Table S5). Furthermore, in line with granular depletion of platelets previously reported in CKD patients,^8^ we detected a reduced platelet content of c-c motif ligand 5 (CCL5), a chemokine stored in α-granules (Figure 5D), although platelet PF4 levels were unaltered (Supplemental Figure S8C). Moreover, we observed a reduced overall granule count in CKD platelets (Figure 5E). Detailed information on the granule type most affected by CKD was not available within this study.

In summary, CKD patients reveal increased plasma levels of the platelet collagen receptor GPVI and a decreased platelet content of granules and CCL5, suggesting prior platelet activation in vivo.

### Improved thrombus formation capacity in CKD by in vivo platelet protection with ASA

**Figure 6:**
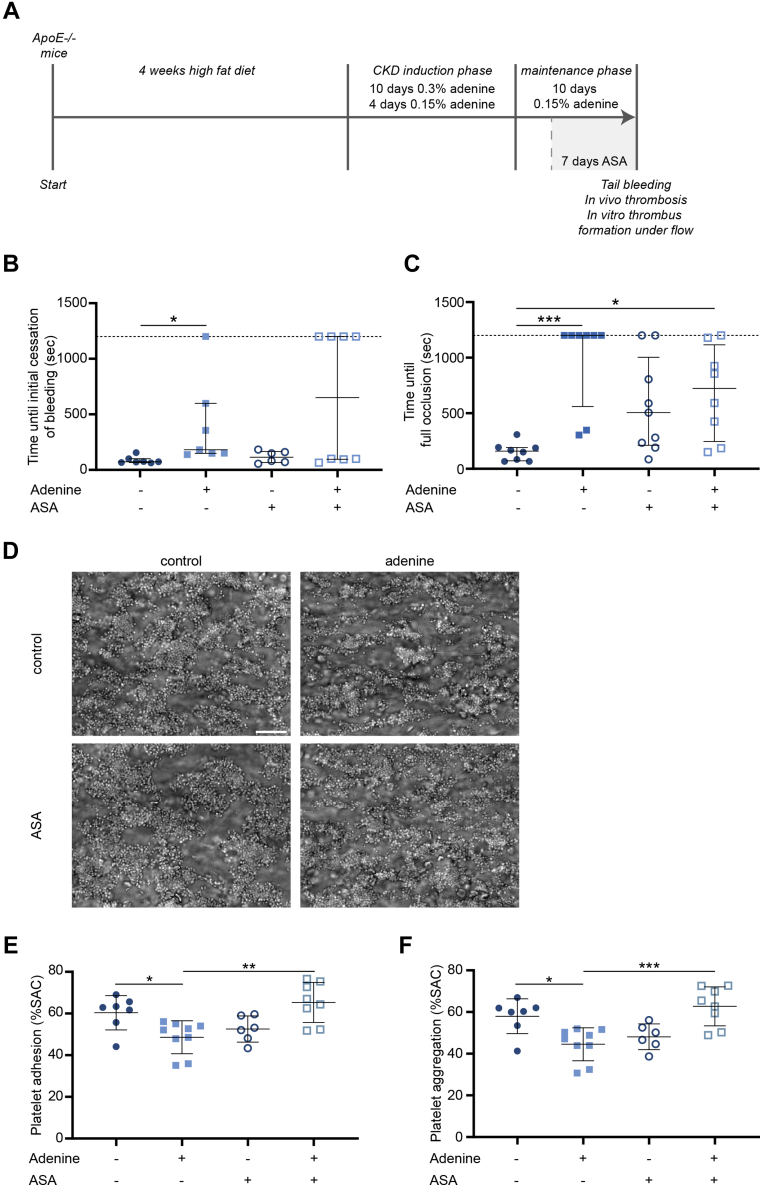
Adenine-Fed Mice Present With Prolonged Bleeding Time, an Impaired Thrombotic Response In Vivo and Reduced In Vitro Thrombus Formation Under Flow ASA treatment restores the adenine-induced impairment of in vitro thrombus formation. (A) Schematic representation of the experimental set-up. (B) Bleeding was examined using the tail bleeding assay in which the time until the initial cessation of bleeding was monitored. n = 6-8 per group. (C) The in vivo thrombotic response was investigated upon a topical FeCl_3_ application to arteries in the cremaster muscles. Time to full vessel occlusion was recorded. n = 8-9 per group. (B and C) When bleeding had not stopped (B) or the vessel was not occluded (C) at the end of the 20-minute analysis time, values were put to the maximum of 1,200 seconds. Values are median with 25th-75th percentiles. ∗*P* < 0.05; ∗∗∗*P* < 0.001 (Kruskal-Wallis test with Dunn’s post hoc test, outlier identification using the ROUT method). (D-F) PPACK anticoagulated mouse whole blood was perfused over a collagen type I surface at 1,000 s^−1^ for 4 minutes. (D) Representative images of platelet adhesion and aggregation. Scale bar = 20 μm. Quantification of the surface area covered by adhered (E) and aggregated (F) platelets. Values are mean ± SD. ∗*P* < 0.05; ∗∗*P* < 0.01; ∗∗∗*P* < 0.001 (1-way analysis of variance with Šídák’s post hoc test). n = 6-9 per group. Abbreviations as in Figures 1 and 2.

Given the unexpected reduction in platelet dysfunction in ex vivo thrombus formation in blood from CKD5D patients on ASA compared with those without, we next studied mice with CKD, induced by an adenine-rich diet, that received ASA or vehicle over the last 7 days (Figure 6A). ASA-mediated platelet inhibition was confirmed by suppressed serum thromboxane B2 levels (Supplemental Figure S9). CKD mice exhibited significantly increased levels of serum creatinine and urea as well as increased levels of kidney fibrosis (Collagen I and αSMA protein), kidney inflammation (*Tnfα, Ccl2, IL1β* gene expression) and kidney injury (Lipocalin-2 [*Lcn2*] expression),^17^ without significant differences between groups without vs with ASA treatment (Supplemental Figure S10). No significant differences in platelet counts were observed (Supplemental Table S6). CKD mice had a significantly prolonged bleeding time, though without clear effects of ASA treatment (Figure 6B) and without significant correlation with CKD extent in terms of kidney fibrosis (Supplemental Figures S11A to S11C). In line with the observed bleeding phenotype, thrombosis induced by a topical FeCl_3_ application led to a significant prolongation of vessel occlusion time in CKD mice compared with control mice (Figure 6C). Also, CKD mice on ASA displayed an increased vessel occlusion time compared with untreated control mice, though with more mice reaching vessel occlusion within the 20-minute timeframe of analysis compared with CKD mice without ASA (6/8 vs 2/8 mice) (Figure 6C). No overall correlation of time to occlusion in relation to CKD extent could be detected (Supplemental Figures S11D and S11E). Parallel ex vivo thrombus formation assays on collagen under flow revealed a significantly decreased platelet adhesion and aggregation in CKD mice (Figures 6D to 6F). Although ASA treatment of control mice led to an expected, although not significant, reduction in platelet adhesion and aggregation, we again observed in conditions of kidney damage a better preservation in thrombus formation capacity upon ASA treatment (Figures 6D to 6F), in agreement with our patient data.

In summary, CKD mice show an increased bleeding time and a delay in injury-induced vessel occlusion, along with a reduced thrombus formation capacity under whole-blood flow ex vivo, the combination pointing to an impaired platelet function in CKD. A delayed vessel occlusion in vivo was also observed in ASA-treated (CKD and non-CKD) mice compared with control mice. However, in CKD mice, similarly as in patients, in vivo ASA treatment partially protected from a CKD-associated decrease in ex vivo thrombus formation capacity in non-coagulating conditions.

## Discussion

Studying platelet function in patients with moderate-to-severe CKD as well as in a CKD mouse model, this study demonstrates signs of prior platelet activation in plasma and platelet content in advanced CKD with a partially reduced secondary platelet response to mainly collagen.

The impaired response of CKD platelets to collagen receptor stimulation as observed by flow cytometry in both whole blood and isolated platelets is in agreement with prior reports.^5^ The partial dysfunction of CKD platelets to collagen receptor stimulation became even more evident when we studied whole-blood thrombus formation on collagen under flow, an assay that is more physiological compared with flow cytometry and which we previously showed to be able to reveal alterations in platelet function that could not be identified by static flow cytometry analyses.^18^ Overall, this revealed the formation of smaller and fewer thrombi with progressing CKD. The reduced ability of thrombus formation in CKD was confirmed in mice with adenine-induced CKD, which presented with a reduced in vitro thrombus formation capacity and an in vivo prolongation of bleeding time and time to vessel occlusion. Our in vivo data are in line with the findings of Makhloufi et al^19^ detecting reduced thrombotic tendency in 5/6 nephrectomy and adenine-induced CKD. Instead, the study of Ortillon et al^20^ pointed at platelet hyperactivation and accelerated thrombus formation upon 5/6 nephrectomy. Potentially a differential degree of induced vascular damage may underlie these differences, with an increased contribution of thrombin, TF and TF-induced hypercoagulation to the increased prothrombotic response observed by Ortillon et al,^20^ whereas collagen and platelet GPVI increasingly contribute to FeCl_3_-induced thrombus formation upon more severe vascular damage,^21^ which may be presented by the hyperlipidemic *ApoE* knockout mice used in our study to mimic the increased proinflammatory, dyslipidemic condition of many CKD patients.^22^

Although the platelet count was significantly reduced in CKD5D patients, this was not to such an extent that it would affect platelet surface area coverage, fibrinogen binding, or PS exposure in the whole-blood thrombus formation assays, as demonstrated previously.^23^ Also, the reduction in erythrocytes was not responsible for the decline in thrombus formation in CKD5D. On the contrary, healthy blood treated with a uremic toxin mixture at advanced CKD concentrations did mimic the reduction in ex vivo thrombus formation in advanced CKD, while causing spontaneous platelet aggregation. This suggested a platelet activation potential by the uremic toxin mix, with reduced secondary platelet responses in ex vivo thrombus assays. Interestingly, 2 of the added uremic toxins—indoxyl sulfate and kynurenic acid−were previously shown to have strong prothrombotic effects in vivo.^24^, ^25^, ^26^ In addition, uremic toxins trigger proinflammatory responses in white blood cells, like, monocytes, with, for example, indoxyl sulfate triggering TNFα secretion from monocytes ^27^ and an uremic toxin mix (including indoxyl sulfate) shown to increase the binding of inflammatory monocytes to platelets.^28^ An additional effect of uremic toxin–stimulated white blood cells on platelet preactivation could thus have contributed to the observed impact of uremic toxin supplementation on platelet responses in whole blood ex vivo, which was not observed upon platelet reconstitution with CKD plasma. Nonetheless, in blood of non–dialysis-dependent CKD patients, no significant correlation was observed for indoxyl sulfate with parameters of ex vivo platelet adhesion or aggregation. Also, homocysteine, another uremic toxin previously described to be prothrombotic,^5^ only correlated weakly with P-selectin expression under flow. Combined, this suggests that in CKD patients the investigated uremic toxins have either indirect platelet effects or that other factors contribute more strongly to the observed platelet phenotype. This could include other uremic toxins accumulating in the blood of CKD patients, increased vascular inflammation, innate immune activation, as well as dialysis-induced platelet activation.^4^^,^^10^ Additionally, the effect of CKD on bone marrow homeostasis and thus megakaryocytes could potentially contribute to the dysfunctional phenotype of platelets in CKD.^29^

Although our observation of impaired thrombus formation suggests at first sight a CKD-mediated inhibition of platelet function that fits the hemorrhagic phenotype, the increase in plasma soluble GPVI and 11-dehydro TXB_2_ levels, and the decreased platelet granule numbers and granule protein content of CCL5 in CKD rather point to prior platelet activation. Given the limited ability of platelets for self-renewal, this can render platelets desensitized or exhausted towards a secondary stimulus. In vivo plasma markers of platelet activation will be elevated in such cases, whereas secondary in vitro platelet reactivity will be decreased. Why PF4 levels were decreased in CKD5 patients on dialysis remains unclear. Considering the immediate effects of dialysis and heparin administration on PF4 levels,^30^, ^31^, ^32^ potentially, exposure to dialysis over a longer period might alter plasma PF4 levels. Exhausted platelets have previously been detected in patients who suffered a stroke or in cancer patients with solid tumors.^33^ Overall, impaired collagen receptor GPVI-mediated platelet activation in advanced CKD due to prior platelet preactivation, as evidenced in our study, is in line with previous observations that platelets initially triggered with the GPVI-ligand CRP-XL resulted in a reduced secondary platelet adhesion on collagen under flow.^34^ A preactivation via GPVI could be due to increased fibrotic responses (and thus collagen content) in patients with advanced CKD^35^ or even increased collagen fragments in plasma of these patients due to enhanced collagen turnover.^36^ Furthermore, fibrin is an agonist of GPVI^37^ and could thus further contribute to prior in vivo platelet stimulation over GPVI in advanced CKD, which is characterized by a hypercoagulable state with increased levels of D-dimers.^4^^,^^38^ In addition, the increased levels of sGPVI might be the result of fibrin-induced GPVI shedding as seen in other thrombotic conditions,^39^ or from platelet pre-activation by uremic toxins, chronic low-grade inflammation and/or endothelial activation in CKD.^4^^,^^10^ In follow-up studies, it would be interesting to determine platelet GPVI surface expression and its relation to sGPVI levels, platelet reactivity, and thrombosis risk.

Unexpected was the observation that CKD5D patients on ASA showed partial protection from platelet dysfunction ex vivo. Platelet adhesion and aggregation capacity under flow was better preserved in CKD5D patients on ASA compared with those not on ASA, with similar data observed in our CKD mouse model. In comparison, patients with CVD but with healthy kidney function did not display a reduced thrombus formation capacity ex vivo nor an impact of ASA treatment. Combined—and keeping in mind that the mere presence of ASA does not impact thrombus formation in this whole-blood flow assay—these data support a concept of prior preactivation with partial secondary functional impairment of platelets in patients with advanced CKD, which is partially mitigated in patients on ASA treatment. Of note, also in CKD5D ASA patients, platelet aggregation and fibrinogen binding responses still remained significantly lower than those in healthy control subjects. Furthermore, platelet-dependent fibrin formation in coagulating conditions was impaired in both CKD5D and CKD5D ASA patients relative to healthy control subjects, and also plasma levels of sGPVI, as well as platelet integrin activation and P-selectin exposure upon GPVI stimulation, were not significantly different between CKD5D and CKD5D ASA patients. Overall, this implies a multifactorial influence of CKD on platelet function as well as of ASA on CKD-associated dysfunction. In terms of ASA effects on cardiovascular risk in CKD patients, current literature is still nondecisive, with findings going from no effect^40^ to beneficial^41^^,^^42^ or even harmful effects^43^ in specific subgroups of CKD patients.^44^ Based on the need for randomized controlled trials to evaluate the effect of low-dose ASA treatment for primary prevention of cardiovascular risk and potential impact on bleeding in specifically CKD patients, the large, randomized ATTACK (Aspirin to Target Arterial Events in Chronic Kidney Disease) trial^45^ was initiated, with study completion estimated in 2025.

### Study limitations

The number of included CKD5D patients treated with or without ASA is relatively low due to our strong predefined exclusion criteria for study eligibility. Also, we could not provide further details on age, sex, and renal function of the healthy control subjects, since the control group was anonymized after blood sampling according to our ethics approval. Therefore, a sex-based analysis was not possible. Similarly, for the mouse model, a sex-based analysis was not possible as only male mice were included in the study. Finally, the studied mouse model induces a moderate degree of CKD (with an ∼2.4-fold increase in both serum creatinine and urea, and an ∼5-fold increase in kidney fibrosis in terms of Collagen I). It displays an increased bleeding risk and—similar to what we observed in both CKD4 and CKD5D patients—a significantly decreased platelet adhesion and aggregation in ex vivo thrombus formation assays but is nonetheless not fully comparable to renal failure and CKD5D in patients.

## Conclusions

We provide evidence that CKD conditions trigger prior platelet preactivation with a secondary partial functional impairment, which increases with CKD severity. Thereby, our paper may contribute to the clarification of the complex thrombotic and hemorrhagic phenotype that is seen in many CKD patients.

### Data availability statement

Data are available from the corresponding author upon reasonable request.

Perspectives **COMPETENCY IN MEDICAL KNOWLEDGE:** CKD is a progressive disease characterized by a reduced kidney function, with patients with complete kidney dysfunction requiring dialysis. Importantly, CKD patients have an imbalance in hemostasis, resulting in an increased risk of thrombotic and hemorrhagic complications. The function of platelets, key mediators of hemostasis, has been shown to be impaired in CKD, although studies have been conflicting. By combining platelet phenotyping and thrombus formation studies in CKD patients and animal models, we provide evidence for an inherent effect of advanced CKD towards pre-activation of circulating platelets, resulting in a partial, secondary impairment of platelet responses to mainly collagen. These secondary impairments in platelet function are less pronounced in CKD patients who are treated with acetylsalicylic acid.

**TRANSLATIONAL OUTLOOK:** CKD patients are at high thrombotic as well as bleeding risk. Current antithrombotic guidelines have not been optimized for this patient population and first require a deeper understanding of platelet (dys)function in CKD. Our study provides an important contribution to that. Moreover, our observations towards the effects of acetylsalicylic acid treatment, both in human and murine samples, might relate to high on treatment platelet reactivity, a phenomenon that is increased in CKD.

## Funding Support and Author Disclosures

This work was financially supported by the Alexander von Humboldt Foundation (to Dr Baaten); the Dutch Heart Foundation (2020T020, to Dr Baaten); the START-Program of the Faculty of Medicine of the RWTH Aachen University (105/20 to Drs Baaten and Noels), the German Heart Foundation/German Foundation of Heart Research (F/61/23, Dr Baaten), the Interdisciplinary Centre for Clinical Research within the faculty of Medicine at the RWTH Aachen University (PTD 1-11 to Dr Kahles and 1-12 to Dr Noels) and by the German Research Foundation (DFG) Project-ID 322900939 SFB/TRR219 (S-03, C-01, C-04, C-05, M-05 and M-07 to Drs, Noels, Kramann, Marx, Floege, Kahles, Speer, and Schunk), Project-ID 403224013 – SFB 1382 (A-04 to Drs Jankowski and Noels) and Project-ID 520275106 Emmy Noether Research Group (Dr Kahles). Dr Henning received a Kaltenbach Stipendium from the Deutsche Herzstiftung. Further funding was provided by the “Else Kröner-Fresenius-Stiftung” (Project 2020_EKEA.60 to Dr Noels, and 2022_EKES.03 to Dr Saritas) and the German Centre for Cardiovascular Research (DZHK-B23Ex to Dr Noels). Dr Boor was supported by the German Research Foundation (DFG, Project IDs 322900939, 454024652, 432698239, and 445703531), European Research Council (ERC Consolidator Grant No 101001791), and the Federal Ministry of Education and Research (BMBF, STOP-FSGS-01GM2202C). Dr Kahles was additionally funded by the European Research Area Network on Cardiovascular Diseases (ERA-CVD and BMBF, Grant No. JTC-2019, MyPenPath - 01KL2004) and the European Foundation for the Study of Diabetes (EFSD)/Novo Nordisk Foundation (NNF2OSA0066111). Dr Jankowski also reports funding from COST Action PerMedik, CA21165, supported by COST (European Cooperation in Science and Technology). Dr Kurt has served as a speaker for SphingoTec/14-4 Pharmaceuticals; and received travel support from 14-4 Pharmaceuticals and Novo Nordisk. Drs Jankowski, Marx, and Noels are founding shareholders of AMICARE Development GmbH. Dr Kahles has served as a speaker for Novo Nordisk, Lilly, AstraZeneca, and DGK-Akademie; consulted for Novo Nordisk, Bayer, and PricewaterhouseCoopers/Strategy; and received travel support from Amgen, Novo Nordisk, Boehringer Ingelheim, Bayer, SphingoTec/14-4, and Lilly. All other authors have reported that they have no relationships relevant to the contents of this paper to disclose.
