# Supplementary material for: Impaired Secondary Platelet Response in Chronic Kidney Disease as a Consequence of Prior Platelet Activation
Source: JACC Basic Transl Sci. 2025 Aug 5;10(9):101355. doi: 10.1016/j.jacbts.2025.101355 (PMC12346058; doi:10.1016/j.jacbts.2025.101355)
Supplement: Supplemental Material [file mmc1.docx]

Impaired secondary platelet response in chronic kidney disease as consequence of prior platelet activation

Constance C.F.M.J. Baaten^1,2,3*^, Julia Wollenhaupt^1^, Tobias M. Henning^1^, Sonja Vondenhoff^1^, Jonas R. Schröer^1^, Eleni Stamellou^4,5^, Turgay Saritas^4,6^, Berkan Kurt^7^, Leonard Boger^4^, Alessandra Antwerpen^7^, Juliane Hermann^1^, Magdolna Nagy^2^, Marieke Sternkopf^1^, Eva Miriam Buhl^8,9^, Ute Raffetseder^4^, Paola E.J. van der Meijden^2,10^, Marijke J. E. Kuijpers^2,10^, Henri M. H. Spronk^2,10,11^, Stefan J. Schunk^12^, Joachim Jankowski^1,3,13^, Danilo Fliser^12^, Thimoteus Speer^14^, Peter Boor^8,9^, Rafael Kramann^4,6^, Florian Kahles^7^, Jürgen Floege^4^, Nikolaus Marx^3,7^, Heidi Noels1,2,3*

^1^Institute for Molecular Cardiovascular Research (IMCAR), University Hospital RWTH Aachen; Aachen, Germany.

^2^Department of Biochemistry, Cardiovascular Research Institute Maastricht, Maastricht University; Maastricht, the Netherlands.

^3^Aachen-Maastricht Institute for Cardiorenal disease (AMICARE), Aachen, Germany. ^4^Department of Nephrology and Clinical Immunology, University Hospital RWTH Aachen; Aachen, Germany.

^5^Department of Nephrology, University Hospital of Ioannina, Ioannina, Greece.

^6^Department of Internal Medicine, Nephrology and Transplantation, Erasmus Medical Center; Rotterdam, the Netherlands.

^7^Department of Internal Medicine I, University Hospital RWTH Aachen; Aachen, Germany.

^8^Institute of Pathology, University Hospital RWTH Aachen; Aachen, Germany.

^9^Electron Microscopy Facility, University Hospital RWTH Aachen; Aachen, Germany. ^10^Thrombosis Expertise Center, Heart and Vascular Center, Maastricht University Medical Center+; Maastricht, the Netherlands.

^11^Department of Internal Medicine, Maastricht University Medical Center+; Maastricht, the Netherlands.

^12^Department of Internal Medicine IV, Nephrology and Hypertension, Saarland University, Homburg/Saar, Germany.

^13^Department of Pathology, Cardiovascular Research Institute Maastricht, Maastricht University Medical Centre; Maastricht, the Netherlands.

^14^Department of Internal Medicine IV, Goethe University Frankfurt, Frankfurt am Main, Germany.

Running title: Platelet dysfunction in chronic kidney disease

Supplementary file

*Shared corresponding authors:

Dr. Constance Baaten, and Dr. Heidi Noels, Institute for Molecular Cardiovascular Research (IMCAR), University Hospital Aachen, RWTH Aachen University, Pauwelsstraße 30, 52074 Aachen, Germany: [cbaaten@ukaachen.de;](mailto:cbaaten@ukaachen.de) [hnoels@ukaachen.de;](mailto:hnoels@ukaachen.de) Tel.: +49-241-8037147.

# Supplementary Materials and Methods

*Materials*

Apyrase was from Sigma Aldrich (Darmstadt, Germany), synthetic cross-linked collagen-related peptide (CRP-xl) was from Cambcol (University of Cambridge, Cambridge, United Kingdom) and FeCl3 was from Honeywell Fluka (Charlotte, NC, USA). PPACK was purchased at Abcam (Cambridge, United Kingdom); dalteparin was from Pfizer (New York, NY, USA); Thrombin Receptor Activating Peptide-6 (TRAP-6; SFLLRN) from Bachem (Bubendorf, Switzerland); unfractionated heparin from Leo Pharma (Ballerup, Denmark) and ASA and iloprost (ilomedine) from Bayer (Berlin, Germany). Tissue factor (Innovin) came from Siemens (Erlangen, Germany). Collagen I was from Nycomed Pharma (Berlin, Germany), while 2-methyl thioadenosine diphosphate trisodium salt (2MeADP) was from Tocris (Wiesbaden-Nordenstadt, Germany). The Thromboxane B2 ELISA kits came from Cayman Chemical (Ann Arbor, MI, USA), the ELISA kits for PF4, GPIbα and GPVI were purchased at ThermoFisher Scientific (Waltham, MA, USA) and the ELISA kit for CCL5 was purchased from Invitrogen by Thermo-Fisher Scientific (Waltham, MA, USA). The DyLight 488-conjugated anti- GPIbβ antibody was from Emfret Analytics (Eibelstadt, Germany). Alexa-Fluor (AF)647- or AF546-labeled fibrinogen and AF350-labeled annexin A5 came from Invitrogen by Thermo- Fisher Scientific (Waltham, MA, USA), PE-conjugated anti-P-selectin antibody was from e- Bioscience (Dreieich, Germany). Fluorescein isothiocyanate (FITC)-conjugated PAC-1 antibody against active integrin αIIbβ3, allophycocyanin (APC)- conjugated anti GPIbα antibody, FITC-conjugated anti-human P-selectin were from Becton-Dickinson Bioscience (Franklin Lakes, NJ, USA). The APC-conjugated anti-CD63 antibody was from Invitrogen Molecular Probes (Waltham, MA, USA). DiOC6 was purchased at Tebu-bio (Heerhugowaard, The Netherlands).

*Sample collection and analysis*

Sample collection and analysis was performed by independent departments and with patient pseudo-anonymization to safeguard data privacy, thereby also reducing potential sources of bias. Blood was collected via venipuncture of the antecubital vein using a 21G needle (Sarstedt, Nümbrecht, Germany) into S-monovette tubes containing either K3-EDTA or 3.2%(w/v) citrate (Sarstedt, Nümbrecht, Germany). Patients on hemodialysis donated blood at the end of the longest interdialytic interval, just before the start of the hemodialysis session and before the administration of heparin. From these patients, blood was drawn via the arteriovenous fistula. A whole blood cell count in EDTA-anticoagulated blood was obtained using a Sysmex XN350 analyzer (Kobe, Japan). Citrated blood samples were immediately used to measure platelet activation and reactivity using flow cytometry and thrombus formation under flow. Alternatively, plasma was prepared and stored at -80°C until analysis.

*Preparation of washed platelets, erythrocytes, and plasma*

Platelet-rich plasma (PRP) was prepared by centrifuging citrate anticoagulated blood at 250 g for 15 min at room temperature (RT). Platelet-poor plasma was prepared by centrifuging EDTA or citrate anticoagulated blood twice at 2150 g for 10 min at RT. Plasma was stored at -80° C until further processing.

Platelets were isolated as described before.(1) In brief, PRP was supplemented with 1:10 v/v acidic citrate dextrose (ACD: 80 mM trisodium citrate, 52 mM citric acid and 180 mM glucose) and subsequently centrifuged at 2200 g for 2 min. Supernatant was discarded and platelets were resuspended in Hepes buffer pH 6.6 (10 mM Hepes, 136 mM NaCl, 2.7 mM KCl, 2 mM MgCl2, 0.1% glucose, 0.1% bovine serum albumin (BSA) and 0.1 U/ml apyrase). After addition of 1:15 v/v ACD, platelets were centrifuged again at 2200 g for 2 min. The washed platelets were then resuspended in Hepes buffer pH 7.45 (10 mM HEPES, 136 mM NaCl, 2.7 mM KCl, 2 mM MgCl2, 0.1% glucose, 0.1% BSA). The platelet count was determined using a Sysmex XN350.

To reconstitute PRP, isolated platelets from healthy volunteers or CKD patients were resuspended in pooled plasma (3.2%(w/v) citrate-anticoagulated) from healthy volunteers or patients with CKD stage 4 to the original platelet count in PRP.

Erythrocytes were isolated by centrifuging citrate-anticoagulated blood at 250 g for 15 min after which the erythrocyte layer was resuspended into Hepes buffer pH 7.45 containing

0.1 U/ml apyrase. Erythrocytes were then centrifuged at 2150 g for 10 min and again resuspended in Hepes buffer pH 7.45 and centrifuged. To increase hematocrit levels of CKD patient blood, 50 µl packed autologous CKD erythrocytes or healthy O^-^ erythrocytes were added to 500 µl whole blood. To reconstitute whole blood and normalize hematocrit values, 450 µl packed erythrocytes were added to 550 µl reconstituted PRP.

*Uremic toxin preparation*

In order to study the influence of uremic toxins on platelet activation and thrombus formation, seven of the most upregulated uremic toxins were pooled and pre-diluted in citrate anticoagulated plasma to prevent short-term local high toxin concentrations and thus potential instant unphysiological effects of the uremic toxin mix directly after addition of the uremic toxin mix to the blood: Phenylacetic acid (3.5 mM f.c.), indoxyl sulphate (37 μg/ml f.c.), hippuric acid (0.6 mM f.c), kynurenic acid (150 μg/l f.c.), p-cresyl sulphate (23 mg/l f.c.), methylguanidine (226 μg/l f.c.) and guanidinosuccinic acid (3 mg/l f.c.). Final concentrations in the experiments reflect blood levels of uremic patients listed in the Eutox database.(2)

*Flow cytometric analysis of platelet activation*

Platelet activation was assessed either in whole blood or in a suspension of washed platelets (50x10^6^/ml) using flow cytometry. To determine platelet activation in whole blood, 3.2% citrate anticoagulated blood was prediluted 1:5 in Hepes buffer pH 7.45. Subsequently, the diluted whole blood was activated with 0.5-5.0 μg/ml CRP-xl, 0.01-0.5 μM 2MeADP or 15-

150 μM TRAP-6 for 15 min. Integrin αIIbβ3 activation and P-selectin expression were

determined by labeling with a FITC-conjugated PAC-1 antibody (0.5 μg/ml) or a PE-conjugated anti P-selectin antibody (4 μg/ml), respectively. Platelets were gated from whole blood by labeling with an APC-conjugated anti-GPIbα antibody.

To assess platelet activation in a suspension of isolated platelets, washed platelets were activated with 1.0-5.0 μg/ml CRP-xl, 0.5-5.0 μM 2MeADP or 7.5-30 μM TRAP-6 in the presence of 2 mM CaCl2 for 15 min. Here, integrin αIIbβ3 activation, P-selectin and CD63 expression were determined by labeling with a FITC-conjugated PAC-1 antibody (1.25 μg/ml), a PE-conjugated anti P-selectin antibody (5 μg/ml) and an APC-conjugated anti CD63 antibody (1.125 μg/ml) respectively. Samples were measured in duplicate with a FACS Canto II (BD, Franklin Lakes, NJ, USA) and analyzed using FlowJo software v.10.6.1 (Ashland, OR, USA).

*Whole blood thrombus formation under flow*

Thrombus formation in human blood under flow was determined basically as described before.(3,4) In short, washed glass coverslips were coated with a 1.0 μl microspot of 100 μg/ml collagen type I, blocked with 1% BSA and mounted in a parallel-plate flow chamber. Then, citrate-anticoagulated whole blood, reconstituted blood or uremic toxin-treated blood supplemented with 7.5 μg/ml AF546-conjugated fibrinogen was recalcified (7.5 mM CaCl2, 3.75 mM MgCl2 f.c.) in the presence of 40 μM PPACK (to inhibit thrombin generation) and perfused over the collagen surface for 3.5 min at 1000 s^-1^. After blood perfusion, thrombi were labeled for P-selectin expression and phosphatidylserine (PS) exposure by perfusing with Hepes buffer pH 7.45 (10 mM HEPES, 136 mM NaCl, 2.7 mM KCl, 2 mM MgCl2, 0.1% glucose, 0.1% BSA) supplemented with FITC-conjugated anti-P-selectin antibody (1:80) and Annexin AF350 (1:200) for 2 min. Alternatively, to allow thrombin generation and fibrin formation, glass coverslips were coated with microspots containing 50 μg/ml collagen type I with 500 pM tissue factor (TF, Innovin). Here, citrate-anticoagulated whole blood supplemented with 7.5 μg/ml AF546-conjugated fibrinogen was recalcified by a 1:10 co-perfusion with coagulation buffer

(Hepes buffer pH 7.45, 63 mM CaCl2, 32 mM MgCl2) and perfused over the coverslip for 10 min at 1000 s^-1^.

To examine thrombus formation under flow in murine blood, washed glass coverslips were coated with a 1.0 μl 100 μg/ml collagen type I microspot and blocked with 1% BSA. PPACK/UFH/dalteparin anticoagulated mouse blood was then perfused over the collagen surface for 3.5 min at 1000 s^-1^.

Bright field and fluorescence images were captured using an EVOS microscope (ThermoFisher Scientific, Waltham, MA, USA), equipped with a 60x oil objective. Image analysis was performed using Fiji.(5) Thrombus formation under flow was described by surface area coverage of adhered platelets (adhesion), aggregated platelets (aggregation), platelets expressing P-selectin, having bound fibrinogen or exposing PS. Platelet aggregation under flow was defined by the surface covered by aggregates, whereas platelet adhesion refers to the surface covered by all adhered platelets regardless of whether they were incorporated into an aggregate, as was also done previously.(6) Fibrin generation under flow was described as the surface area covered by fibrin.

*Plasma markers of platelet activation and electron microscopy*

Plasma levels of Platelet Factor 4 (PF4), glycocalicin and soluble GPVI were determined in EDTA-anticoagulated plasma using commercially available ELISA kits (ThermoFisher Scientific: EHPF4 (1:1000 plasma predilution), EH91RB (1:20 plasma predilution) and EH230RB (1:10 plasma predilution) respectively) according to the manufacturer’s instructions. Plasma levels of 11-dehydro TXB2 were assessed in citrate- anticoagulated plasma using a commercially available ELISA kit (Cayman chemical: 519510) according to the manufacturer’s instructions.

For electron microscopy, isolated platelets were fixed in 3% glutaraldehyde in 0.1 M Soerensen’s phosphate buffer (Merck, Darmstadt, Germany) for 24h and embedded in 2.5% low-melting agarose (Sigma, Steinheim, Germany). After post-fixing with 1% OsO4 (Roth,

Karlsruhe, Germany) in 25 mM sucrose buffer (Merck, Darmstadt, Germany) the samples were dehydrated by ascending ethanol series (30, 50, 70, 90 and 100%) for 10 min each. Last step was repeated 3 times. Dehydrated samples were incubated in a mixture of Epon resin (Serva, Heidelberg, Germany) and ethanol (1:1) for 1h and finally in pure Epon for 1h. Samples were embedded in fresh Epon and polymerized at 90°C for 2h. Ultrathin sections (90 nm) were stained with 0.5% uranyl acetate and 1% lead citrate (both EMS, Munich, Germany) to enhance contrast. Samples were examined using a transmission electron microscope (Zeiss Leo906, Oberkochen, Germany) operating at an acceleration voltage of 60 kV.

*Uremic toxin plasma concentrations*

Levels of indoxyl sulfate and homocysteine were quantified in citrate anticoagulated plasma. Homocysteine concentrations were measured using a commercially available fluorometric assay (Abcam: Ab228559). The levels of indoxyl sulfate were determined using mass spectrometry. To this end, citrated plasma samples (500 µl) were deproteinated using perchloric acid and subsequently fractionated by reversed-phase chromatography (LiChroprep RP-18 40-63 µm, Merck) using 0.1% TFA in water (Fisher Scientific, Pittsburgh, USA) as solvent A and 90% ethanol in water as solvent B. A stepwise gradient ran as follows: 0–21 min 0% B; 21 – 28 min 20% B; 28–35 min 40% B; 35-42 min 60% B; 42–50 min 100% B. The

separation and elution were monitored with UV absorbance at λ 230, 272, 280, and 495 nm, and collected in a 3.5 ml interval. The resulting fractions were pooled appropriately and concentrated using the freeze-drying technique (Thermo Fisher Scientific, Langerwehe, Germany). Subsequently, the sample was resolved in 10 µl water; 1 µl sample was pipetted directly onto the (MALDI) target plate (MTP-Ground steel 400/384; Bruker Daltonics, Bremen, Germany) using a–cyano-4-hydrocinnamic acid as MALDI matrix. The subsequent mass- spectrometric analyses were performed using '*Magnetic Resonance Mass Spectrometry*' (MRMS) (ScimaX^TM^; Bruker Daltonics, Bremen, Germany). Mass spectra were acquired in the negative ion mode and a mass range of 50 to 1000 Dalton. Measurements were generated at

30 scans per measurement and a frequence of 4 MHz. Signals were normalized to the total ion count.

*Mice*

All animal experiments were approved by the local authorities (Landesamt für Natur, Umwelt und Verbraucherschutz Nordrhein-Westfalen, Germany, approval number 81- 02.04.2017.A504), were based on a detailed protocol description and with appropriate sample size calculations and complied with the local, national and European Union ethical guidelines. Every effort was made to minimize suffering. Male C57BL/6J apolipoprotein E-deficient (*Apoe*^-^

^/-^) mice, bred and housed under specific pathogen-free conditions in the animal facility of the University Hospital RWTH Aachen, were 10 to 12 weeks of age at the start of the experiments and had access to food and water ad libitum. If applicable, mice were housed in groups of 2-

3. The overall health status of the animals was checked daily and evaluation according to a specific score sheet was done every day. To prevent potential experimental confounders, animals from different breeding cages were divided comparably over different treatment groups. Adenine- nephropathy was induced by feeding the mice a high fat diet (21% fat, 19.5% casein, 0.8% calcium and 0.5% phosphate: Altromin, Lage, Germany) for 4 weeks after which they received a high fat diet supplemented with 0.3% adenine for 10 days followed by a high fat diet supplemented with 0.15% adenine until the end of the experiment 14 days later, as described previously*.*(7) In parallel, control mice were fed the high fat diet without adenine supplementation. To examine effects of platelet inhibition, control and adenine-fed mice were treated with acetylsalicylic acid administered via the drinking water (75 mg/l acetylsalicylic acid) during the last 7 days of the experiment with daily refreshment of drinking water. At the end of the experiment, mice were anesthetized by an intraperitoneal injection of 100 mg/kg bodyweight ketamine and 10 mg/kg xylazine. To study thrombus formation under flow, blood was collected into 40 µM PPACK, 5 U/ml unfractionated heparin and 40 U/ml dalteparin by retro-orbital puncture. Investigators were blinded for the experimental conditions. A complete

blood cell count using a Celltac Alpha Vet hematology analyzer (Nihon Kohden, Rosbach, Germany) was made of blood drawn on K3- EDTA (Sarstedt, Nümbrecht, Germany). To prepare serum, blood collected into Serum Gel Z tubes (Sarstedt, Nümbrecht, Germany) was left to clot and was subsequently centrifuged twice at 2000 g for 10 min. Serum was stored at

-80°C for the assessment of creatinine and urea levels. Serum thromboxane B2 (TXB2) levels were determined using a commercially available ELISA kit (Cayman Chemical, 501020) according to the protocol provided by the manufacturer.

*In vivo thrombosis and tail bleeding*

Thrombosis was assessed upon FeCl3-induced damage of the arterial vascular bed within the cremaster muscle. Anesthesia was induced by an intraperitoneal injection of 100 mg/kg body weight ketamine and 10 mg/kg xylazine and maintained until the end of the experiment. A total of 0.1 μg Dylight X488 anti GPIbα IgG derivative per gram body weight was injected intravenously in the lateral tail vein to visualize platelets. Next, the cremaster muscle was dissected free from the surrounding tissue and stretched. Using a filter paper, a 5%(w/v) FeCl3 solution was topically applied for 1 min followed by a quick rinse of the muscle with sterile 0.9% saline. The thrombosis process was monitored for 20 min after FeCl3 application using a Leica DM6 FS microscope, equipped with a 20x objective (Leica, Wetzlar, Germany). Time until complete vessel occlusion was recorded by two independent researchers in a blinded manner. Animals from different experimental groups were analyzed in mixed order to prevent potential confounders of order of measurements.

*Tail bleeding*

Bleeding was examined using the tail bleeding model. In short, the distal tip (2 mm) of the tail was amputated and the tail was submerged into prewarmed saline (0.9% NaCl, 37°C). Bleeding was monitored over a period of 20 minutes. Animals from different experimental groups were analyzed in mixed order to prevent potential confounders of order of

measurements. The investigators monitoring bleeding time were not informed of the experimental conditions. When bleeding had not stopped by then, a total bleeding time of 20 minutes was recorded.

*Tissue protein isolation and western blot analysis*

Tissue homogenates from explanted kidneys were prepared in pre-cooled 300 µl RIPA lysis and extraction buffer (ThermoFisher Scientific, Germany) supplemented with a protease inhibitor cocktail (cOmplete^TM^, Roche) and phosphatase inhibitor cocktail (PhosSTOP, Sigma) using steel beads in combination with a tissue lyzer (TissueLyzer LT, Qiagen, Germany). After final centrifugation at 14000g for 5 min at 4°C to remove solid debris, the supernatant was collected, and total protein content was quantified by using the DC protein assay (BioRad, Germany). Samples were stored at -20°C until further processing. 30 µg of protein per lane were subjected to sodium dodecyl sulfate-polyacrylamide gel electrophoresis (SDS-PAGE) followed by transfer to a nitrocellulose membrane (Amersham Protran, Fisher Scientific, Germany) via semi-dry blotting (Trans-Blot SD, BioRad, Germany). Membranes were blocked with 5% (w/v) non-fat dry milk in TBS or 5% (w/v) BSA in TBS (20 mM Tris, 150 mM NaCl, pH 7.5) for 1h while gently shaking at RT, washed (TBS-T, several washing steps) and incubated with the respective primary antibody overnight at 4°C according to the manufacturer’s instructions. The following antibodies were used: collagen 1 (COL1, 1310-01, Southern Biotech, USA) and α-smooth muscle actin (αSMA, ab124964, Abcam, UK). Glyceraldehyde 3- phosphate dehydrogenase (GAPDH, #5174, Cell Signaling, USA) was used as internal loading control. After several washing steps with TBS-T, membranes were incubated with species- matched secondary antibody (HRP-conjugated, Cell Signaling, USA) for 1h at RT, followed by final washing steps and chemiluminescent detection using Pierce ECL Western Blotting Substrate (ThermoFisher Scientific, Germany) or SuperSignal™ West Atto Ultimate Sensitivity Substrate (COL1 detection) and the iBright imaging system (ThermoFisher Scientific, Germany). Quantitative analysis was performed using the iBright analysis software

(ThermoFisher Scientific, Germany) by quantifying band intensities with normalization to the

corresponding loading controls (GAPDH). Data are presented as fold-increase in loading control-normalized band intensities relative to the mean value of sham-treated animals.

*Quantitative real-time PCR analysis*

Kidney tissue was transferred into Trizol and homogenized with steel beads in combination with a tissue lyzer (TissueLyzer LT, Qiagen, Germany). After adding chloroform and centrifugation for 15 min at 11 500 g at 4°C the upper aqueous phase was used for further RNA isolation according to the manufacturer’s instructions (RNeasy Micro Kit, Qiagen, Germany) with additional DNase treatment (RNase-free DNase Set, Qiagen, Germany). RNA concentration was measured using a NanoVue^TM^ Plus (VWR, Germany). cDNA generation was performed using 500 ng RNA dissolved in RNase-free water and the SuperScript^TM^ VILO^TM^ Mastermix (ThermoFisher Scientific, Germany) according to manufacturer’s instructions, with increased incubation time at 42°C for 120 min to increase cDNA yield. qPCRs were performed by combining SybrGreen master mix (PowerUp™ SYBR™ Green Master Mix, Applied Biosystems, USA), cDNA and the respective primers and using QuantStudio 3 (Thermo Fisher Scientific, USA). All qPCRs were run in duplicates or triplicates to increase accuracy, after which values were averaged per sample and gene of analysis. Then, mRNA expression was normalized per sample against housekeeping genes (β-actin, GusB, Hprt1). Quantitative analysis was performed using the comparative ΔΔCT method. Graphs represent fold-increase in housekeeping-normalized mRNA expression compared to the mean value of Sham-treated animals. The used SybrGreen primer sequences (Eurofins, Germany) are listed in the following table.

| **Gene Abbreviation** | **Forward primer (5’-XXX-3’)** | **Reverse primer (5’-XXX-3’)** |
| --- | --- | --- |
| *β-actin* | CAC TGT CGA GTC GCG TCC | TCA TCC ATG GCG AAC TGG TG |
| *GusB* | ATAAGACGCATCAGAAGCCG | ACTCCTCACTGAACATGCGA |
| *Hprt1* | GCT TTC CCT GGT TAA GCA GTA CA | GAG AGG TCC TTT TCA CCA GCA A |
| *Tnf* | CCACCACGCTCTTCTGTCTA | AGGGTCTGGGCCATAGAACT |
| *Ccl2* | GCTGTAGTTTTTGTCACCAAGC | GACCTTAGGGCAGATGCAGT |
| *Il1β* | CAACCAACAAGTGATATTCTCCATG | GATCCACACTCTCCAGCTGCA |
| *Lcn2* | GCTGTCGCTACTGGATCAGA | CTGTACCTGAGGATACCTGTGC |

# Supplementary References

1. Baaten CCFMJ, Veenstra LF, Wetzels R et al. Gradual increase in thrombogenicity of juvenile platelets formed upon offset of prasugrel medication. Haematologica 2015;100:1131-1138.
2. The EUTox group. The European Uremic Toxins (EUTox) Database.
3. de Witt SM, Swieringa F, Cavill R et al. Identification of platelet function defects by multiparameter assessment of thrombus formation. Nat Commun 2014;16:4257.
4. Swieringa F, Baaten CCFMJ, Verdoold R et al. Platelet control of fibrin distribution and microelasticity in thrombus formation under flow. Arterioscler Thromb Vasc Biol 2016;36:692-699.
5. Schindelin J, Arganda-Carreras I, Frise E et al. Fiji: an open-source platform for biological image analysis. Nat Methods 2012;9:676-682.
6. Nagy M, van Geffen JP, Stegner D et al. Comparative Analysis of Microfluidics Thrombus Formation in Multiple Genetically Modified Mice: Link to Thrombosis and Hemostasis. Front Cardiovasc Med 2019;6:99.
7. Wollenhaupt J, Frisch J, Harlacher E et al. Pro-oxidative priming but maintained cardiac function in a broad spectrum of murine models of chronic kidney disease. Redox Biol 2022;56:102459.

**Table S1: Characteristics and hematological parameters of patients and healthy controls.** Hematological parameters were assessed in EDTA-anticoagulated blood. Mean ± SD are depicted. * *P* < 0.05; ** *P* < 0.01; *** *P* < 0.001 compared to the healthy control group (one-way ANOVA with Dunnett’s post hoc test). *ASA, acetylsalicylic acid; CVD, cardiovascular disease; eGFR, estimated glomerular filtration rate; Hb, hemoglobin; HD, hemodialysis; MPV, mean platelet volume; n.a., not applicable; n.d., not determined; PLT, platelets; RBC, red blood cells; WBC, white blood cells.*

|  | **CKD3** | **CKD4** | **CKD5 HD** | **CKD5 HD**  **ASA** | **CVD** | **CVD ASA** | **Healthy**  **Controls** |
| --- | --- | --- | --- | --- | --- | --- | --- |
| N | 24 | 18 | 8 | 11 | 5 | 10 | 24 |
| Age (years) | 54.5 ±  17.8 | 49.5 ±  17.1 | 59.3 ±  13.3 | 63.5 ±  18.0 | 55.4 ±  15.0 | 70.9 ± 6.6 | n.d. |
| Female/male  (n) | 7/17 | 8/10 | 2/6 | 0/11 | 1/4 | 2/8 | n.d. |
| Creatinine  (mg/dl) | 1.76 ±  0.39 | 2.80 ±  0.70 | 8.48 ±  3.01 | 7.02 ±  1.83 | 0.90 ±  0.15 | 0.91 ±  0.17 | n.d. |
| Urea (mg/dl) | 66.79 ±  20.15 | 117.8 ±  51.13 | 108.7 ±  36.73 | 118.5 ±  37.90 | 31.6 ±  10.04 | 37.8 ±  8.88 | n.d. |
| eGFR  (ml/min/1.73 m^2^) | 40.98 ±  9.58 | 23.38 ±  5.14 | <15 | <15 | 91.9 ±  18.6 | 80.3 ±  11.3 | n.d. |
| Dialysis  (years) | n.a. | n.a. | 6.0 ± 5.4 | 4.3 ± 3.9 | n.a. | n.a. | n.a. |
| Diabetes (n) | 8 | 2 | 4 | 4 | 0 | 5 | n.a. |
| CVD (n) | 0 | 0 | 0 | 7 | 5 | 10 | n.a. |
|  | | | | | | | |
| WBC (x10^9^/l) | 8.2 ± 1.9  *** | 7.1 ± 1.9 | 4.8 ± 1.3 | 6.7 ± 1.4 | 7.3 ± 1.4 | 7.0 ± 2.0 | 6.3 ± 1.3 |
| RBC (x10^12^/l) | 4.7 ± 0.6 | 4.1 ±  0.5*** | 3.5 ±  0.5*** | 3.7 ±  0.5*** | 4.9 ± 0.4 | 5.1 ± 0.5 | 4.8 ± 0.3 |
| Hb (g/dl) | 13.6 ± 1.7 | 12.5 ±  1.2** | 11.3 ±  1.4*** | 11.3 ±  1.7*** | 14.9 ± 1.4 | 14.6 ± 2.1 | 14.0 ±  0.9 |
| PLT (x10^9^/l) | 253 ± 63 | 244 ± 65 | 158 ±  67** | 207 ± 78 | 234 ± 53 | 233 ± 67 | 254± 58 |
| MPV (fl) | 10.4 ± 0.9 | 10.5 ± 1.0 | 10.8 ± 0.9 | 10.3 ± 1.2 | 10.9 ± 0.6 | 10.4 ± 0.6 | 9.9 ± 1.2 |

**Table S2: CKD etiology and medication use per CKD group**

| **Patient characteristics** | **CKD3** | **CKD4** | **CKD5 HD** | **CKD5 HD ASA** |
| --- | --- | --- | --- | --- |
| N | 24 | 18 | 8 | 11 |
| ***CKD etiology, N (%)*** | | | | |
| Diabetic nephropathy | 0 (0) | 0 (0) | 2 (25) | 2 (18) |
| Hypertension | 3 (13) | 0 (0) | 0 (0) | 1 (9) |
| Glomerulonephritis^a^ | 10 (42) | 10 (55) | 1 (12.5) | 2 (18) |
| Inherited kidney disease^b^ | 0 (0) | 3 (17) | 1 (12.5) | 1 (9) |
| Other^c^ | 10 (42) | 5 (28) | 4 (50) | 5 (46) |
| Unknown | 1 (4) | 0 (0) | 0 (0) | 0 (0) |
| ***Medication, N (%)*** | | | | |
| Acetylsalicylic acid (aspirin) | 0 (0) | 0 (0) | 0 (0) | 11 (100) |
| Erythropoietin | 0 (0) | 2 (11) | 3 (37.5) | 5 (46) |
| Vitamin D | 15 (63) | 7 (39) | 8 (100) | 7 (64) |
| Antihypertensives | 22 (92) | 17 (94) | 6 (75) | 9 (82) |
| Glucose lowering therapy | 7 (29) | 2 (11) | 1 (12.5) | 3 (27) |
| Lipid lowering therapy | 10 (42) | 7 (39) | 1 (12.5) | 8 (73) |
| Immune suppressants | 6 (24) | 5 (28) | 4 (50) | 2 (18) |

*aGlomerulonephritis, IgA nephropathy, membranous glomerulonephritis, membranous nephropathy*

*bPolycystic kidney disease, Alport syndrome*

*cSjögren’s syndrome, ANCA-associated vasculitis, primary focal segmental glomerulosclerosis, monoclonal gammopathy of renal significance, focal segmental glomerulosclerosis, acute cortical necrosis, hydronephrosis, cardiorenal*

**Table S3: CVD etiology and medication use per CVD group.** *HFmrEF, Heart failure with mildly reduced ejection fraction; HFpEF, Heart failure with preserved ejection fraction; HFrEF, Heart failure with reduced ejection fraction; NSTEMI, Non-ST-elevation myocardial infarction.*

| **Patient characteristics** | **CVD** | **CVD ASA** |
| --- | --- | --- |
| N | 5 | 10 |
| ***CVD etiology, N (%)*** | | |
| Coronary artery disease | 1 (20) | 10 (100) |
| Unstable angina | 0 (0) | 1 (10) |
| Heart failure (HFpEF) | 0 (0) | 3 (30) |
| Heart failure (HFmrEF, HFrEF) | 2 (40) | 2 (20) |
| NSTEMI + Percutaneous coronary intervention | 0 (0) | 3 (30) |
| Coronary venous bypass | 0 (0) | 3 (30) |
| Valvular heart disease^a^ | 3 (60) | 3 (30) |
| ***Medication, N (%)*** | | |
| Acetylsalicylic acid (aspirin) | 0 (0) | 10 (100) |
| Erythropoietin | 0 (0) | 0 (0) |
| Vitamin D | 2 (40) | 1 (10) |
| Antihypertensives | 2 (40) | 8 (80) |
| Glucose lowering therapy | 0 (0) | 4 (40) |
| Lipid lowering therapy | 2 (40) | 8 (80) |
| Immune suppressants | 0 (0) | 1 (10) |

**^a^***Aortic valve stenosis, mitral/aortic valve regurgitation****,*** *aortic valve replacement****,*** *bicuspid aortic valve*

**Table S4: Hematological parameters of patients and healthy controls for the reconstitution experiments in main Fig. 5.** Hematological parameters presented as Mean ± SD. * *P* < 0.05; ** *P* < 0.01; *** *P* < 0.001 compared to the healthy control group (unpaired t- test), *Hb, hemoglobin; MPV, mean platelet volume; PLT, platelets; RBC, red blood cells; WBC, white blood cells.*

| **Patient**  **characteristics** | **CKD4** | **Healthy**  **Controls** |
| --- | --- | --- |
| N | 12 | 5 |
| WBC (x10^9^/l) | 7.1 ± 1.9 | 6.0 ± 0.7 |
| RBC (x10^12^/l) | 3.8 ± 0.5** | 5.1 ± 0.8 |
| Hb (g/dl) | 11.3 ± 1.6*** | 15.2 ± 2.0 |
| PLT (x10^9^/l) | 256 ± 39* | 211 ± 37 |
| MPV (fl) | 10.7 ± 0.9 | 10.1 ± 0.7 |

**Table S5: Plasma levels of homocysteine and indoxyl sulfate do not show a strong correlation to markers of platelet adhesion, aggregation and activation in patients with CKD stage 3 and 4.** Levels of homocysteine and indoxyl sulfate were measured in citrated plasma. Correlations were assessed between uremic toxin levels *vs.* parameters of *ex vivo* thrombus formation (platelet adhesion, aggregation, P-selectin expression, fibrinogen binding and PS exposure), plasma sGPVI levels and plasma levels of 11-dehydro TXB2 using Spearman’s correlation. Shown are Spearman correlation coefficients (r) and corresponding P-values. Significant correlations are printed in italic. CKD3 n= 24, CKD4 n= 18. *PS, phosphatidylserine.*

|  | Homocysteine | | Indoxyl sulfate | |
| --- | --- | --- | --- | --- |
|  | r | P-value | r | P-value |
| Homocysteine |  | | 0.106 | 0.51 |
| Indoxyl sulfate | 0.106 | 0.51 |  | |
| Platelet adhesion | -0.268 | 0.095 | -0.070 | 0.66 |
| Platelet aggregation | -0.263 | 0.10 | -0.094 | 0.56 |
| P-selectin | *-0.338* | *0.033* | -0.218 | 0.17 |
| Fibrinogen binding | -0.086 | 0.60 | 0.057 | 0.72 |
| PS exposure | 0.189 | 0.24 | -0.076 | 0.64 |
| sGPVI | -0.023 | 0.89 | 0.124 | 0.45 |
| 11-dehydro TXB2 | 0.021 | 0.90 | 0.008 | 0.96 |

**Table S6: Renal and hematological parameters of mice fed a high fat diet with or without adenine supplementation.** Creatinine and urea levels were measured in serum. For bodyweight and kidney parameters: Median with 25^th^-75^th^ percentiles, * *P* < 0.05; ** *P* < 0.01,

*** *P* < 0.001 compared to controls; ^§§^*P*<0.01 compared to control + ASA (Kruskal-Wallis test with Dunn’s post-hoc test).

Hematological parameters were assessed in EDTA-anticoagulated blood. Mean ± SD. ^#^ *P* <

0.05 compared to adenine (one-way ANOVA with Holm-Šídák’s post-hoc test).

*ASA, acetylsalicylic acid; PLT, platelets; RBC, red blood cells; WBC, white blood cells.*

|  | **Control** | **Adenine** | **Control +**  **ASA** | **Adenine +**  **ASA** |
| --- | --- | --- | --- | --- |
| Bodyweight (g) | 29.4  [28.4-31.7] | 26.6  [25.9-27.7]*** | 30.9  [29.5-31.6] | 27.5  [26.0-29.9]^§§^ |
| **Kidney parameters** | | | | |
| N | 8 | 8 | 8 | 8 |
| Creatinine (µM) | 13.0  [13.0-16.0] | 31.5  [25.3-42.0]** | 13.0  [13.0-16.8] | 38.5  [25.0-45.8]**/§§ |
| Creatinine/ bodyweight (µM/g) | 0.5  [0.4-0.5] | 1.3  [1.0-1.6]* | 0.4  [0.4-0.6] | 1.5  [0.9-1.7]*/§§ |
| Urea (mM) | 12.9  [11.7-13.4] | 30.8  [25.8-37.8]* | 12.0  [10.4-14.6] | 31.9  [21.6-39.8]*/§§ |
| **Hematological parameters** | | | | |
| N | 6 | 7 | 5 | 7 |
| WBC (x10^9^/l) | 4.5 ± 2.0 | 6.1 ± 2.0 | 4.7 ± 1.8 | 4.5 ±1.6 |
| RBC (x10^12^/l) | 8.1 ± 1.4 | 8.5 ± 2.0 | 8.1 ± 0.6 | 6.6 ± 1.1^#^ |
| PLT (x10^9^/l) | 832 ± 246 | 940 ± 263 | 996 ± 159 | 1037 ± 185 |


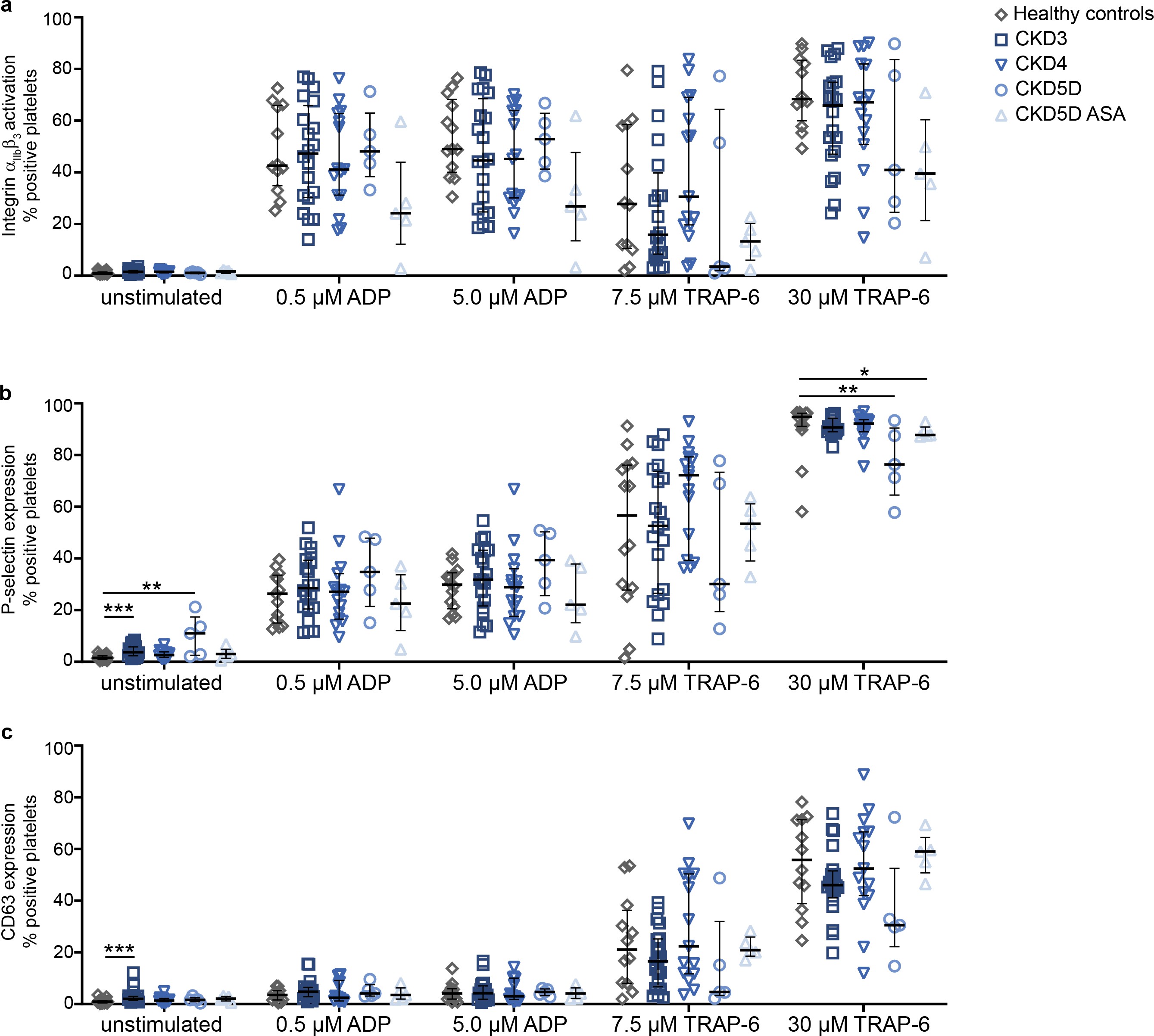


**Figure S1. No significant differences in ADP-induced integrin** α**IIb**β**3 activation and P- selectin expression in CKD platelets, but reduced TRAP-6 induced P-selectin expression in CKD5D platelets.** Isolated platelets (50x10^6^/ml) were left unstimulated or activated with ADP or TRAP-6 in the presence of 2 mM CaCl2 for 15 minutes. Integrin αIIbβ3 activation (**a**), P-selectin (**b**) and CD63 expression (**c**) were assessed using the FITC- conjugated PAC-1 antibody, a PE-conjugated anti-P-selectin antibody or an APC-conjugated anti-CD63 antibody respectively. Data are illustrated as median with 25^th^-75^th^ percentiles. * *P*

< 0.05; ** *P* <0.01; *** *P* <0.001 (Kruskal Wallis test with Dunn’s post-hoc test). Healthy controls n = 13-18, CKD3 n= 20, CKD4 n= 15, CKD5D n= 5 and CKD5D ASA n= 5. *ASA, acetylsalicylic acid; D, hemodialysis.*


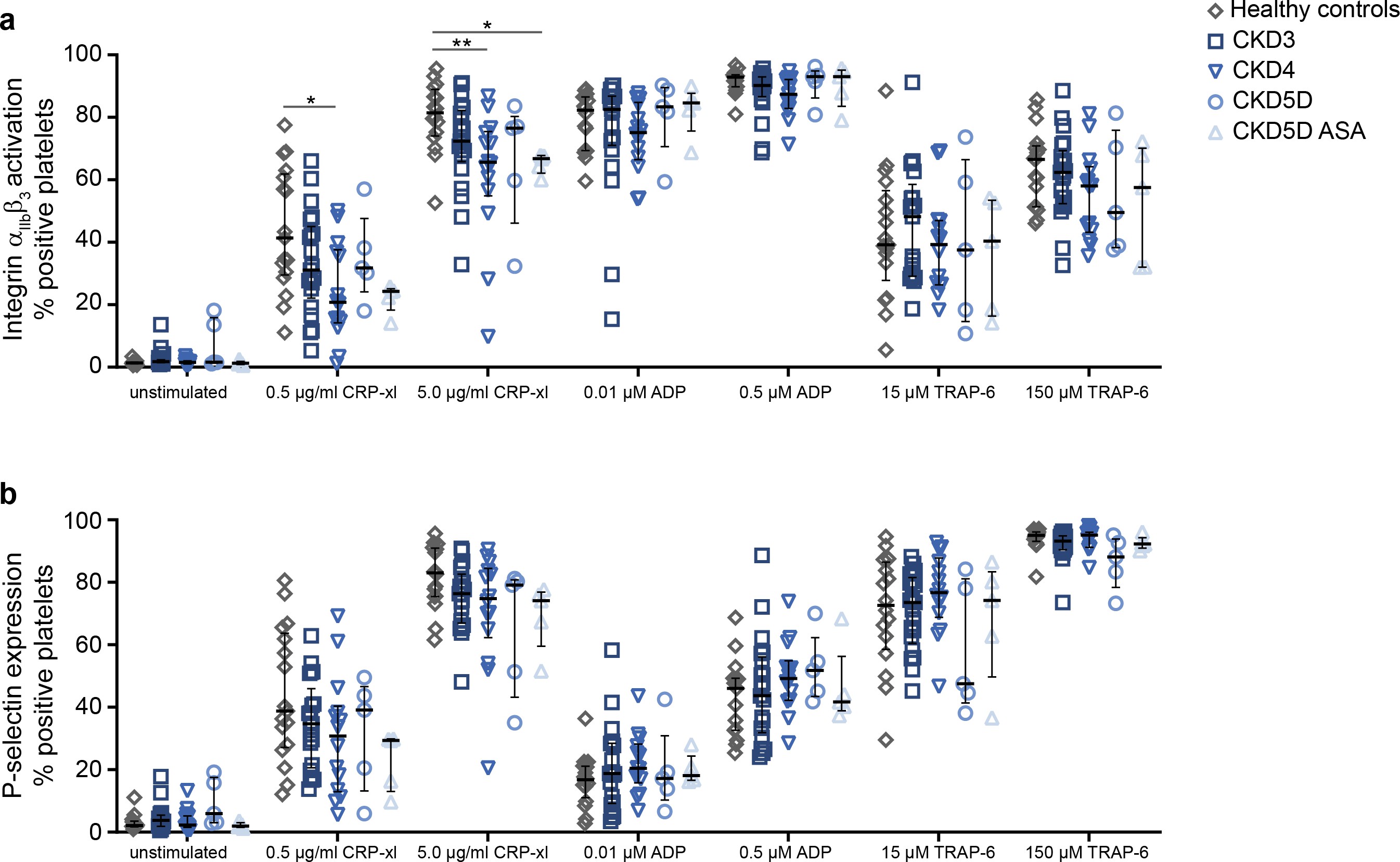


**Figure S2. Depending on CKD stage and strength of platelet stimulus, predominantly CRP-induced integrin activation is impaired in CKD platelets.** Diluted whole blood was either left unstimulated or activated with CRP-xl, ADP, or TRAP-6 for 15 minutes. Within the platelet gate (GPIbα positive events), integrin αIIbβ3 activation (**a**) and P-selectin expression

(**b**) were assessed using the FITC-conjugated PAC-1 antibody or a PE-conjugated anti-P- selectin antibody respectively. Data are illustrated as median with 25^th^-75^th^ percentiles. * *P* < 0.05; ** *P* < 0.01, (Integrin activation - unstimulated, CKD3 vs. healthy controls: *P-value* = 0.069), (Kruskal Wallis test with Dunn’s post-hoc test). Healthy controls n = 17, CKD3 n= 21, CKD4 n=14, CKD5D n=5 and CKD5D ASA n=5. *ASA, acetylsalicylic acid; D, hemodialysis*


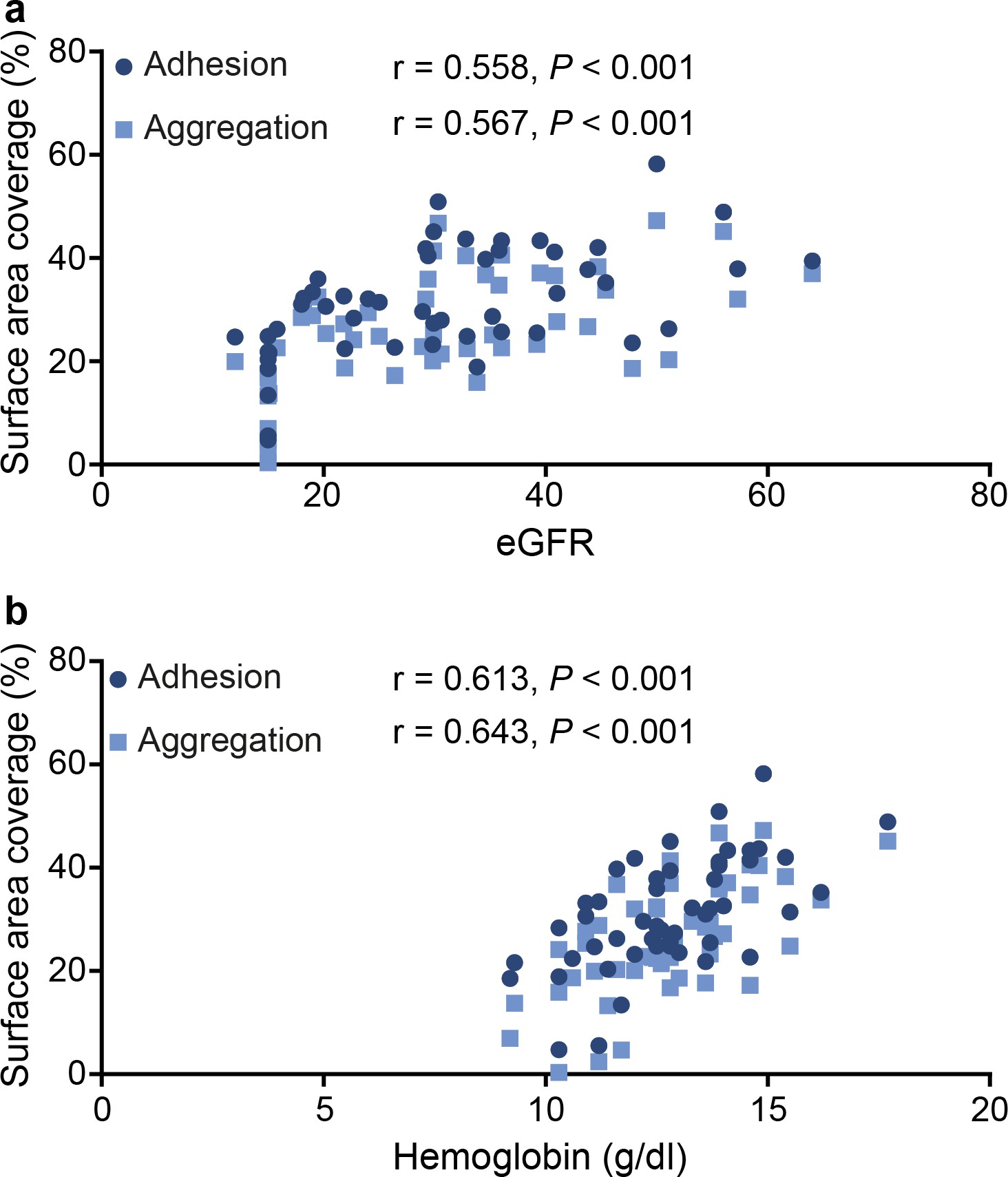


**Figure S3. Thrombus formation under flow shows a moderate to strong correlation to the severity of CKD and anemia.** Shown are dot plots and Pearson correlation coefficients

(r) of thrombus formation under flow expressed as platelet adhesion and aggregation (% surface area coverage) versus eGFR (**a**) and hemoglobin (**b**). Patients taking ASA were excluded from these analyses. The eGFR for dialysis patients was set at 15.


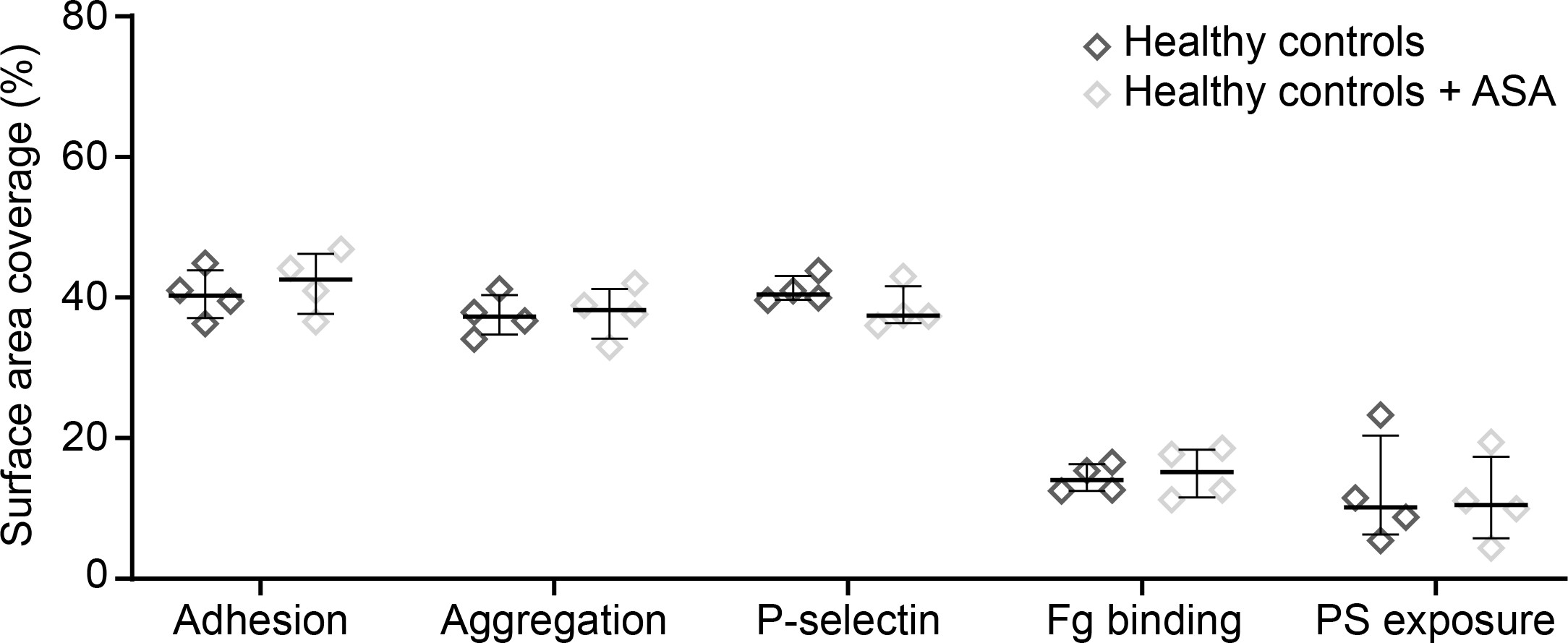


**Figure S4. *Ex vivo* supplementation of acetylsalicylic acid (ASA) does not affect *ex vivo* whole blood thrombus formation.** Citrate anticoagulated whole blood of healthy controls, untreated or with *ex vivo* addition of ASA (100 μM), was recalcified in the presence of PPACK and perfused over a collagen type I surface at 1000 s^-1^ for four minutes. Integrin activation was monitored by determining the amount of bound fluorescent fibrinogen to the thrombi. P-selectin expression and PS exposure were measured by labeling the formed thrombi with an antibody directed against P-selectin and Annexin A5 respectively. Shown is the quantification of the surface area covered by adhered platelets, aggregated platelets, P-selectin positive platelets, bound fibrinogen, and PS positive platelets. Data are depicted as median with 25^th^-75^th^ percentiles. *ASA, acetylsalicylic acid; Fg, fibrinogen; PS, phosphatidylserine.*


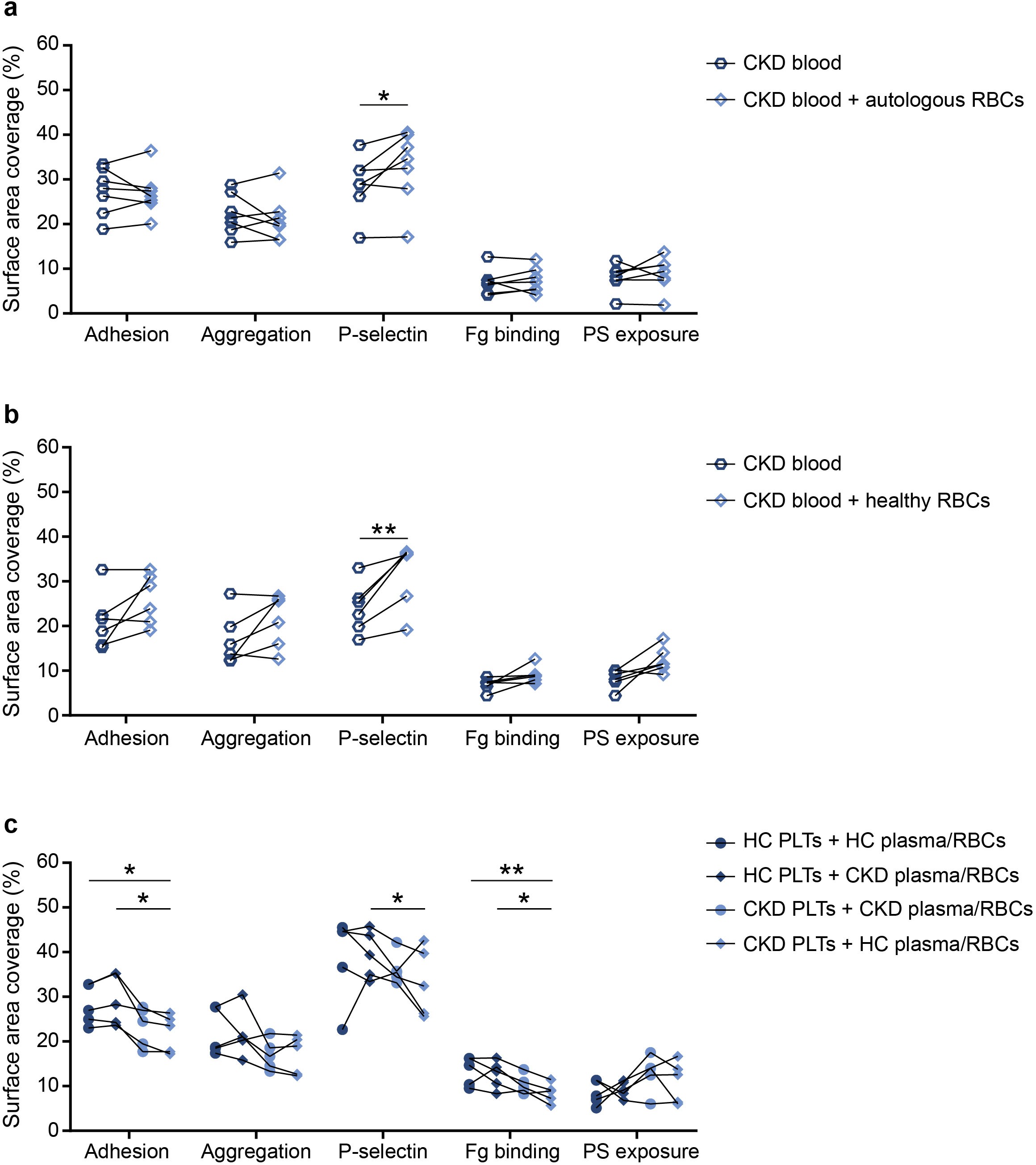


**Figure S5. Neither supplementation of erythrocytes to whole blood nor reconstitution of CKD platelets with healthy plasma and erythrocytes can restore the CKD-associated impairment in thrombus formation.** Citrate anticoagulated whole blood of CKD4 patients was supplemented with washed autologous (**a**) or healthy O^-^ erythrocytes (**b**) which increased hematocrit levels to 121 and 118 percent on average, respectively. Alternatively, platelets from healthy controls or CKD4 patients were resuspended in healthy or CKD4 citrate anticoagulated plasma (Final Plt count: 140 x10^9^/l) and reconstituted with washed packed erythrocytes (Final Hct: 36.4%) (**c**). Thrombus formation under noncoagulating conditions on a collagen type I surface was studied as in Figure 2. * *P* < 0.05; ** *P* < 0.01; (Repeated measures two-way

ANOVA with Šídák’s post-hoc test), n=6-7 (**a, b**) or (Repeated measures two-way ANOVA with Tukey’s post-hoc test), n=4 (**c**). *Fg, fibrinogen; HC, healthy control; PLTs, isolated platelets; PS, phosphatidylserine; RBCs, red blood cells.*


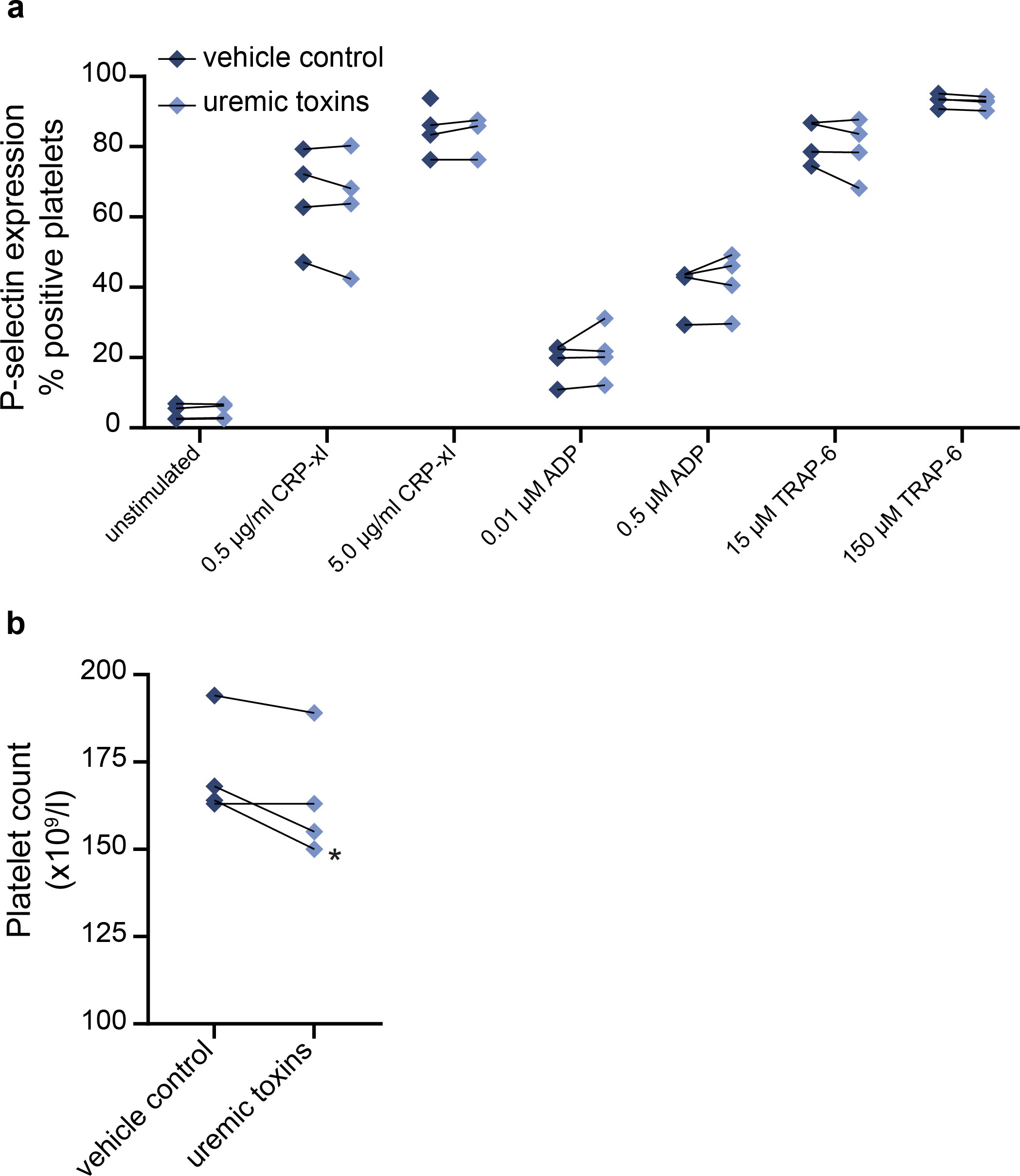


**Figure S6. Uremic toxin mix does not directly affect platelet activation markers in static conditions.** Citrate anticoagulated whole blood of healthy controls was treated with a mixture of phenylacetic acid, indoxyl sulphate, hippuric acid, kynurenic acid, p-cresyl sulphate, methylguanidine and guanidinosuccinic acid, at concentrations reflecting advanced CKD,(2) or alternatively, with vehicle control. **(a)** Platelet activation was measured using flow cytometry by quantifying P-selectin expression. **(b)** Platelet count analyzed via a hematology analyzer.


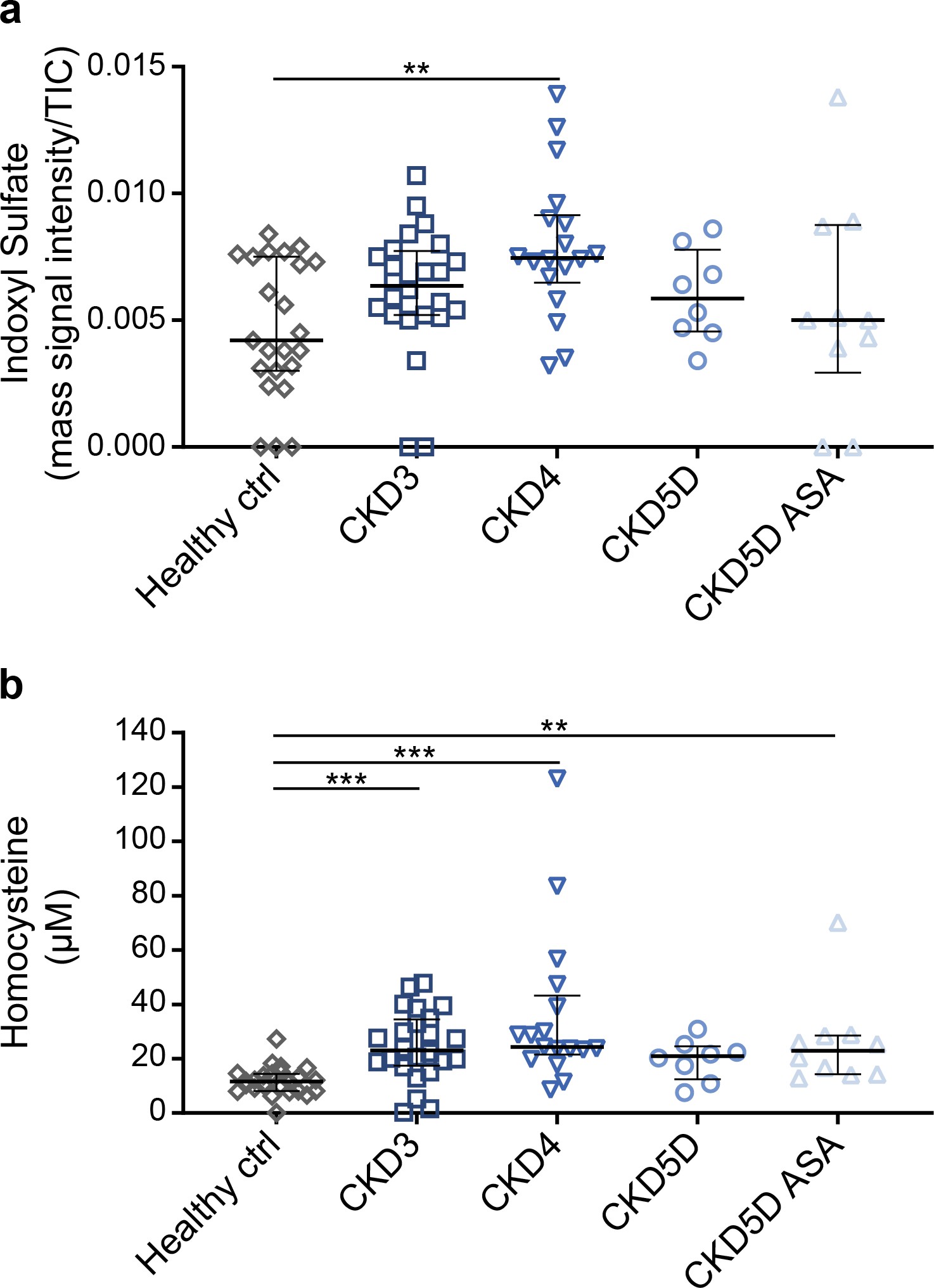


**Figure S7. Accumulation of uremic toxins in CKD.** Plasma levels of indoxyl sulfate (**a**) and homocysteine (**b**) as determined using mass-spectrometric analysis (**a**) or a commercially available fluorometric assay (**b**). Healthy controls n=23-24, CKD3 n=24, CKD4 n=17-18, CKD5D n=8, CKD5D ASA n=10. Data are depicted as median with 25^th^-75^th^ percentiles. ** *P*

< 0.01, *** *P* < 0.001: (Kruskal Wallis test with Dunn’s post-hoc test). *ASA, acetylsalicylic acid; D, hemodialysis; TIC, total ion count.*


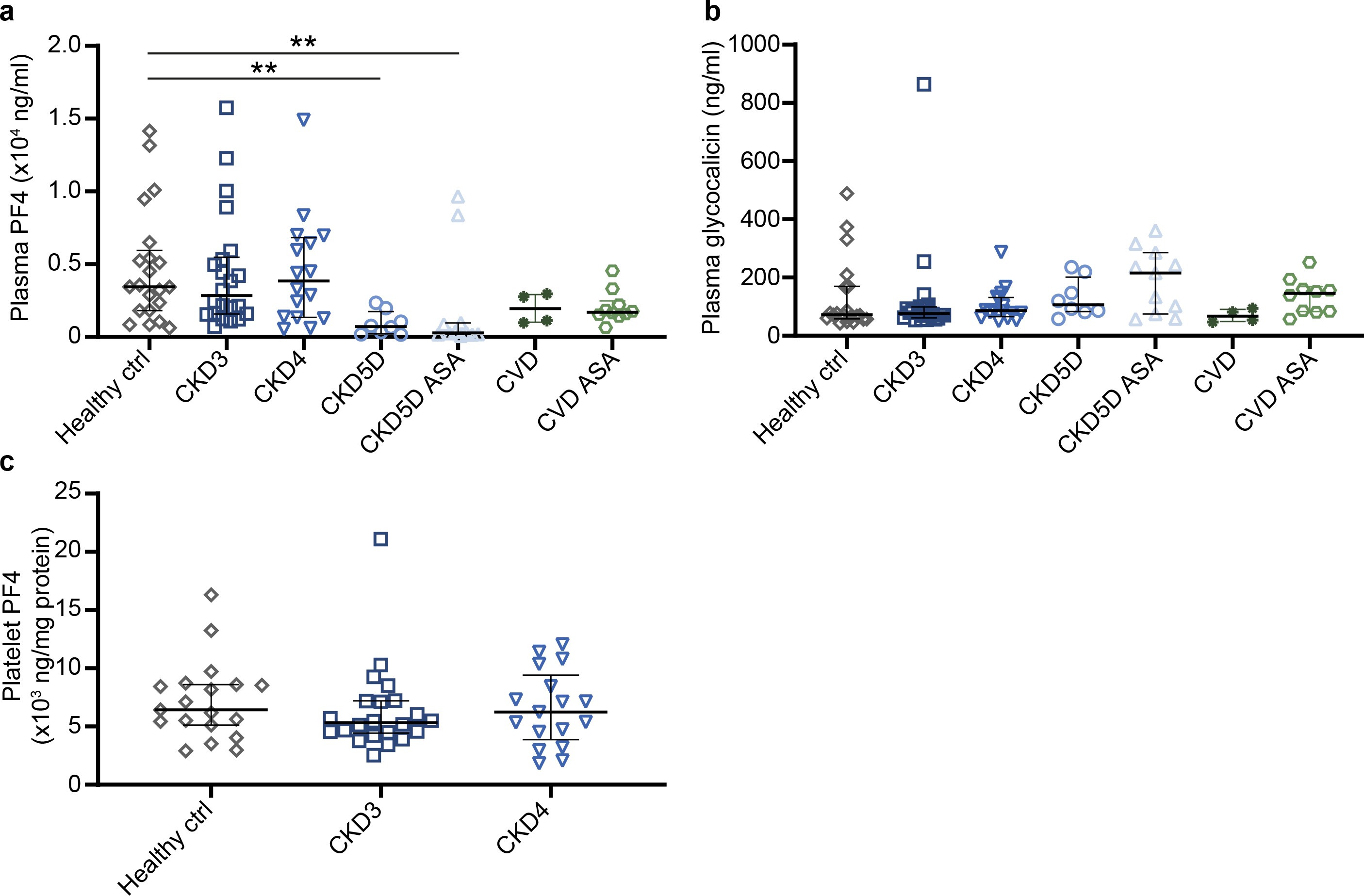


**Figure S8. Plasma levels of PF4 are reduced in CKD5D patients, while glycocalicin levels are unaffected.** Plasma levels of PF4 (**a**) and glycocalicin (**b**) as measured by ELISA. Healthy controls n=21, CKD3 n=22, CKD4 n=16, CKD5D n=8, CKD5D ASA n=11, CVD n=4, CVD ASA

n=10. Platelet levels of PF4 (**c**) as measured by ELISA. Healthy controls n=19, CKD3 n=22, CKD4 n=17. Data are depicted as median with 25^th^-75^th^ percentiles. ** *P* < 0.01 (Kruskal Wallis test with Dunn’s post-hoc test). *ASA, acetylsalicylic acid; D, hemodialysis.*


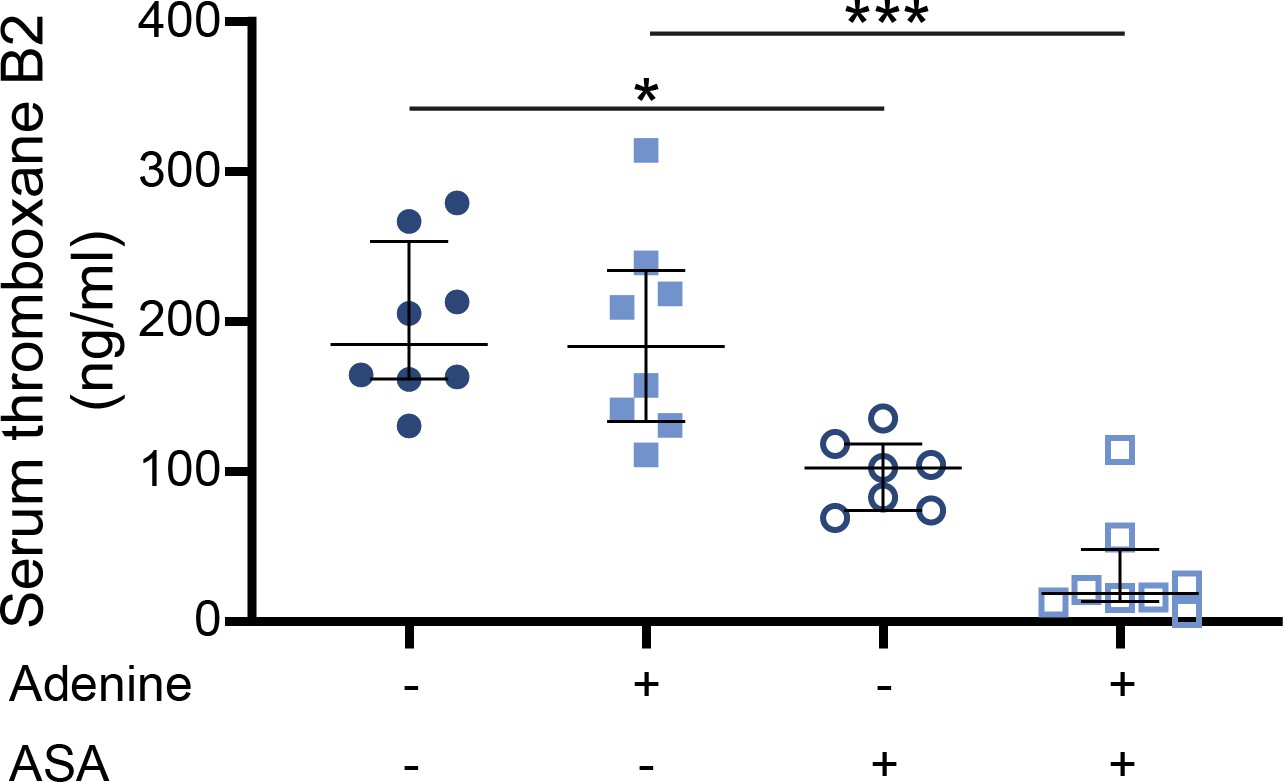


**Figure S9. Administration of acetylsalicylic acid was sufficient to significantly suppress thromboxane formation.** To monitor whether ASA administration in our mouse model was sufficient to inhibit platelet function, serum was collected at the end point and thromboxane B2 levels were measured by ELISA. Data are depicted as median with 25^th^-75^th^ percentiles. * *P* < 0.05; *** *P* <0.001 (Kruskal-Wallis test with Dunn’s post-hoc test). Control mice - ASA: n= 8, Adenine mice - ASA: n=8, control mice + ASA: n= 7 and adenine mice + ASA: n= 8. *ASA, acetylsalicylic acid.*


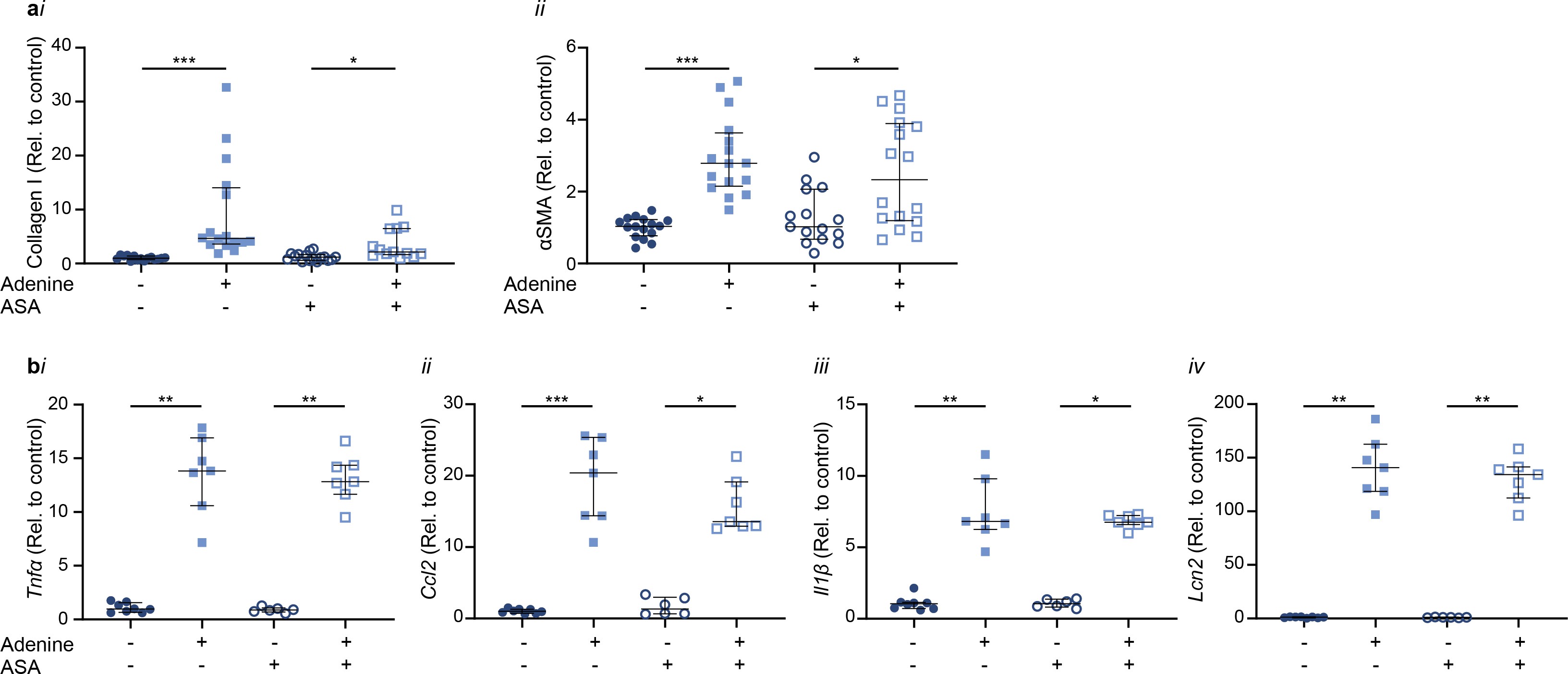


**Figure S10. Adenine-rich diet significantly induced kidney fibrosis and inflammation, without impact of ASA treatment.** Analysis of kidney fibrosis and inflammation in the mouse model depicted in Figure 6. **a)** Protein levels of Collagen I and alpha smooth muscle actin (αSMA) in kidney, as determined by western blot analysis. Normalized to GAPDH and displayed relative to controls. **b)** Gene expression analysis of the inflammatory markers tumor necrosis factor α (*Tnf α*), chemokine *Ccl2* and interleukin-1 β (*Il1β*) and the kidney injury marker lipocalin-2 (*Lcn2*). Normalized to a combination of *β-actin, hprt1* and *gusb,* and displayed relative to controls. **a-b)** Data are depicted as median with 25^th^-75^th^ percentiles. * *P* < 0.05; ** *P* < 0.01; *** *P* <0.001 (Kruskal-Wallis test with Dunn’s post-hoc test). *ASA, acetylsalicylic acid.*


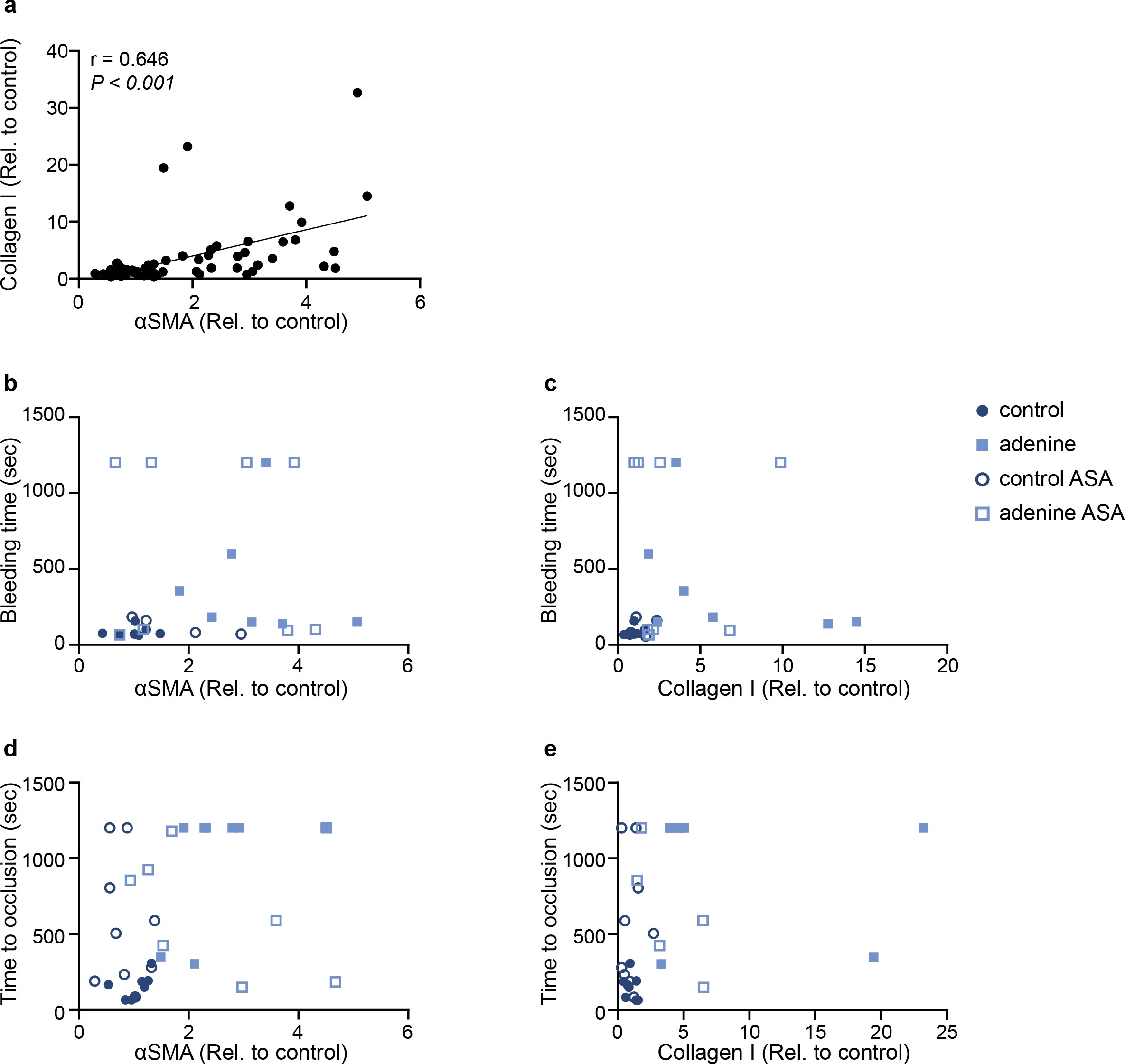


**Figure S11. No overall correlation of kidney fibrosis extent and bleeding or thrombotic parameters.** Analysis of the mouse model depicted in Figure 6. Protein levels of Collagen I and alpha smooth muscle actin (αSMA) in kidney were determined by western blot analysis, normalized to GAPDH and displayed relative to controls. **a)** Significant correlation of the fibrosis markers Collagen I and αSMA in kidney. Linear regression analysis with Spearman correlation coefficient. **b-c)** Bleeding time in relation to kidney fibrosis markers. **d-e)** Time to full occlusion in the *in vivo* thrombosis model in relation to kidney fibrosis markers.
